# Tuberculosis burden attributable to smoking in China 1990–2021 and projections to 2040: A secondary analysis of GBD 2021 data

**DOI:** 10.18332/tid/215180

**Published:** 2026-07-20

**Authors:** Feng Zhao, Tingyong Yan

**Affiliations:** 1 Department of Respiratory and Critical Care Medicine, Huai'an Hospital of Huai'an City, Huai'an City, China

**Keywords:** smoking, tuberculosis, China, Global Burden of Disease

## Abstract

**INTRODUCTION:**

Smoking is a well-established modifiable risk factor for tuberculosis (TB). However, the evolving smoking-attributable TB burden in China remains to be fully characterized. The objective was to examine temporal trends in the smoking-attributable TB burden in China from 1990 to 2021.

**METHODS:**

A secondary analysis was conducted on the 2021 Global Burden of Disease Study data. Data on the number of deaths and disability adjusted life years (DALYs) due to smoking-attributable TB, as well as age-standardized rates, were extracted for the overall Chinese population, males, and females from 1990 to 2021. Joinpoint regression and age-period-cohort analyses described 32-year trends. A Bayesian age-period-cohort (BAPC) model was used to forecast the TB burden from 2022 to 2040. Drivers behind the burden trends were investigated through a decomposition analysis.

**RESULTS:**

Between 1990 and 2021, TB deaths, DALYs, age-standardized mortality rate (ASMR) and age-standardized DALY rate (ASDR) showed significant reductions, with the ASMR and ASDR declining from 5.10/100000 (95% UI: 3.64–6.73) and 158.48/100000 (95% UI: 114.49–203.94) in 1990 to 0.52/100000 (95% UI: 0.37–0.76) and 19.15/100000 (95% UI: 13.85–26.70) in 2021, respectively. Declines in ASMR (-95.26% vs -89.35%) and ASDR (-93.69% vs -87.35%) were larger in females than in males. Age-period-cohort analyses revealed that mortality and DALY rates peaked at ages 30–34 years and then declined. Male mortality increased slightly at ages 65–69 years. Both period and cohort effects demonstrated consistent downward trends. BAPC projections indicated that after 2022, deaths and DALYs would decline initially but rise again, whereas ASMR and ASDR would continue to fall.

**CONCLUSIONS:**

The smoking-attributable TB burden in China exhibits significant age- and sex-specific patterns. Despite overall attenuation, projected rebounds in deaths by 2040 call for sustained public health interventions.

## INTRODUCTION

Tuberculosis (TB) is a chronic infectious disease caused by *Mycobacterium tuberculosis*, transmitted through the air and primarily affecting the lungs^1^. The latest World Health Organization data indicate that in 2023, approximately 10.8 million people developed TB worldwide, resulting in about 1.25 million deaths. In the same year, China’s TB incidence was 52 per 100000 population, less than half the global average, ranking 29th among the 30 high-burden countries. Nevertheless, because of its large population, China still reported an estimated 741000 cases, ranking third globally and accounting for 6.8% of all cases worldwide, trailing only India (26%) and Indonesia (10%)^2^. Despite intensified control efforts in recent years, TB remains a substantial public health challenge in China with the high-to-medium prevalence^3^.

Evidence shows that interventions targeting modifiable risk factors can substantially reduce China’s TB burden. Multiple studies have demonstrated that smoking increases infection risk, accelerates disease progression, and impairs treatment outcomes^4,5^. Smoking compromises pulmonary architecture and suppresses airway immunity, greatly enhancing susceptibility to *Mycobacterium tuberculosis*. Among infected individuals, smokers experience faster disease progression, higher relapse rates, and greater mortality than non-smokers^6,7^. Smoking may also diminish the efficacy of anti-TB therapy and increase the likelihood of treatment failure^8^. Cessation interventions among smokers have been shown to reduce TB recurrence and secondary transmission^9^. Although China has implemented progressive tobacco-control policies in recent years^10^ and overall smoking prevalence is declining, population aging and the emergence of new tobacco products pose fresh challenges to TB prevention and control. The smoking-attributable TB burden may exhibit evolving epidemiological patterns, yet systematic quantification of these trends is lacking.

In light of these considerations, this study utilized the 2021 Global Burden of Disease (GBD) database and applied age-period-cohort modeling to systematically evaluate long-term trends in the TB burden attributable to smoking in China from 1990 to 2021, and to forecast changes from 2022 to 2040. Such evidence was intended to inform more targeted prevention strategies and intervention programs.

## METHODS

### Data source

This study is based on secondary analyses of data from the GBD 2021 study^11^. GBD 2021 systematically collates disease-burden estimates for 204 countries and regions from 1990 to 2021, covering 371 diseases and injuries and 88 risk factors. Since smoking data in the GBD are only available for individuals aged ≥30 years, we extracted the following indicators: 1) deaths and DALYs due to TB attributable to smoking during 1990–2021, for the total population, males, and females, aged ≥30 years, together with age-standardized mortality rates (ASMR), age-standardized DALY rates (ASDR), and their percentage changes; and 2) deaths and DALYs by age group, crude mortality rates (CMR) and crude DALY rates (CDR). Each rate was estimated using the GBD methodology and presented with a 95% uncertainty interval (UI). To compute the UIs, 1000 random draws were performed at each computational step, integrating uncertainties from multiple sources, including variability in input data, measurement error corrections, and estimates of residual non-sampling error. The UI was defined as the range between the 25th and 97.5th percentiles of the ordered sample values.

In the GBD 2021 study, TB was defined according to the International Classification of Diseases (ICD) codes. Specifically, ICD-10 codes A10-A14, A15-A18.89, A19-A19.9, B90-B90.9, K67.3, K93.0, M49.0, N74.0-N74.1, P37.0, and U84.3 were used^12^.

### Statistical analysis

*Descriptive analysis*

We described temporal trends in smoking-attributable TB in China between 1990 and 2021 for the total population and separately for males and females, as well as age-specific trends within the total population.

*Joinpoint regression analysis*

Joinpoint regression model is commonly used to detect inflection points in time-series data. Within a log-linear regression framework, it identifies one or more calendar time points that divide the series into segments with distinct linear trends. In our research, five jointpoints were applied. The rate of change within each segment is expressed as the annual percentage change (APC), while the overall trend across the entire period is summarized by the average annual percentage change (AAPC). A trend is deemed statistically significant if the AAPC/APC differs from zero with p<0.05, with the Bonferroni correction applied to maintain the overall asymptotic significance level. In this study, we applied sex-stratified Joinpoint regression to ASMR and ASDR for smoking-attributable TB in China, and presented their AAPCs and APCs. Joinpoint software (v5.2.0.0) was used for both model construction and visualization.

*Estimated annual percentage change (EAPC)*

The EAPC was used to quantify the average trend change over a specific time period. In a log-linear regression model characterized by the equation:

*Y= α+ βX+ ε*

where *Y* = ln (ASMR/ASDR) and *X* = calendar year. The EAPC was determined by the formula 100 ×(exp(β)-1), with its 95% confidence interval (CI) derived from the model parameters. If both the EAPC and its 95% CI were above or below zero, the ASMR or ASDR was considered to have significantly increased or decreased over time. A trend was deemed statistically non-significant when the 95% CI included zero. Furthermore, the EAPCs between males and females were compared using the Z-test method. All analyses were conducted in R (v4.3.0).

*Age-period-cohort model*

We used age-period-cohort models to examine long-term trends in smoking-attributable TB death and DALY rates in China, disentangling the effects of age, calendar period and birth cohort on the disease burden. The age effect reflects differences in mortality and DALY rates across age groups. The period effect captures temporal influences affecting all age groups. The cohort effect represents variations across successive birth cohorts. The APC model can be expressed as:

*Y*=log (APC)= *μ* + *α_a_* + *β_p_* + *γ_c_* + ϵ

Log(rate) represents the natural logarithm of the mortality or DALY rate; μ represents the intercept, indicating the basic level of outcomes; *α_a_*, *β_p_* and *γ_c_* represent the age, period and cohort parameters, respectively; and ϵ is the random error.

Five-year intervals were used: Given the absence of smoking data for the population aged <30 years in the GBD 2021 database, the grouping of age started at 30–34 years and ended with the oldest at ≥95 years, yielding 14 age groups in total. Calendar period was split into six intervals: 1992–1996, 1997–2001, …, 2017–2021. Birth cohorts were defined by 19 successive five-year bands: 1895–1899, 1900–1904, …, 1985–1989. For model parameter estimation, the median value within each dimension served as the reference, which were aged 60–64 years, period 2002–2006, and birth cohort 1940–1944, respectively.

Age-period-cohort modeling outputs include: longitudinal age curves, which depict age-specific rates for the reference cohort after adjusting for period bias, illustrating the age effect on the disease burden trend; period and cohort relative risks (RRs), based on the reference group and the age-specific rates, which quantify the strength of period and cohort effects on smoking-attributable TB trends; net drift, the overall temporal trend of death and DALY rates expressed as percentage change per year (%/year); local drift, which reflect age-specific temporal changes of death and DALY rates, expressed as %/year. Age-period-cohort online tool (https://analysistools.cancer.gov/apc/) was used for analyses.

*Bayesian age-period-cohort (BAPC) projection*

The BAPC model extends the age-period-cohort framework by integrating Bayesian theory. By assigning appropriate prior probability distributions to age, period and cohort effects to carry out probabilistic modeling, it resolves the parameter-identification dilemma inherent in traditional age-period-cohort models, where collinearity among age, period, and cohort induces non-identifiability of parameter estimation. The model employs a second-order random walk prior. This prior assumes that second-order differences follow a zero-centered normal distribution, encouraging smooth, gradual evolution over time. The corresponding precision parameters (inverse variances) are assigned a weakly informative Gamma hyperprior, Gamma (1; 0.0005). The model imposes smoothing constraints on the effect parameters through a well-specified prior structure, while leveraging Markov chain Monte Carlo (MCMC) algorithms to simulate the posterior distribution and estimate parameters numerically. This retains inherent data information while damping random fluctuations, yielding robust forecasts when dealing with calendar periods. To enhance computational efficiency and stability, the BAPC model in the study incorporated the Integrated Nested Laplace Approximation (INLA), effectively circumventing the poor mixing and convergence problems commonly encountered in conventional Bayesian methods. Previous research has shown that this approach offers superior predictive coverage and accuracy^13^. We fitted the BAPC model in R (v4.3.0) using the *INLA* (v23.09.09) and *BAPC* (v0.0.36) packages. Age-specific population data for 1990–2021 and population projections for 2022–2040, together with the GBD world-standard age structure, were used to generate sex-specific forecasts of smoking-attributable TB burden in China over the next 19 years. The BAPC projected deaths, DALYs, ASMR, and ASDR were validated in a sensitivity analysis using the Nordpred method.

*Decomposition analysis*

The decomposition analysis was conducted to quantify the contributions of population growth, population aging, and epidemiological changes to the changes in deaths and DALYs due to smoking-attributable TB in China from 1990 to 2021. This analysis was conducted in R (v4.3.0) with the method developed by Das Gupta. Disease burden was decomposed in the overall Chinese population, as well as separately for males and females.

## RESULTS

### Temporal trends in the smoking-attributable TB burden in China, 1990-2021

Table 1 summarizes changes in deaths, DALYs and corresponding age-standardized rates (ASRs) for smoking-attributable TB in the total population and by sex, in China from 1990 to 2021. All indicators, deaths, DALYs, ASMR and ASDR, declined markedly in the total population, in males and in females.

**Table 1:** Deaths, DALYs and their ASRs for smoking-attributable TB in the total population, males and females, China, 1990 and 2021

*Sex*	*Deaths*		*Percent* *change*	*ASMR per 100000*		*Percent* *change*
	*1990 (95% UI)*	*2021 (95% UI)*		*1990 (95% UI)*	*2021 (95% UI)*	
**Both**	44157 (31422–58171)	10845 (7712–15895)	-75.44	5.10 (3.64–6.73)	0.52 (0.37–0.76)	-89.84
**Male**	40689 (28322–53781)	10395 (7287–15404)	-74.45	9.76 (6.86–12.95)	1.04 (0.74–1.55)	-89.35
**Female**	3468 (2430–4777)	451 (287–677)	-87.00	0.88 (0.60–1.21)	0.04 (0.03–0.06)	-95.26
	***DALYs***		***Percent*** ***change***	***ASDR per 100000***		***Percent*** ***change***
	***1990 (95% UI)***	***2021 (95% UI)***		***1990 (95% UI)***	***2021 (95% UI)***	
**Both**	1512817 (1087072–1953859)	399118 (288525–556172)	-73.62	158.48 (114.49–203.94)	19.15 (13.85–26.70)	-87.92
**Male**	1411709 (998625–1832715)	383792 (276095–540736)	-72.81	295.59 (209.51–384.54)	37.39 (26.91–52.7)	-87.35
**Female**	101107 (71639–139432)	15326 (10255–22686)	-84.84	22.71 (16.10–31.50)	1.43 (0.96–2.10)	-93.69

DALYs: disability-adjusted life years. ASRs: age-standardized rates. TB: tuberculosis. ASMR: age-standardized mortality rate. ASDR: age-standardized DALYs rate. UI: uncertainty interval.

Specifically, the total number of deaths fell from 44157 to 10845 (a 75.44% reduction) with deaths in males dropping from 40689 to 10395, and deaths in females decreasing from 3468 to 451. ASMR also declined continuously, with the reduction among females (-95.26%) substantially larger than that among males (-89.35%). Reductions of DALYs and ASDR exceeded 70% and 85%, respectively, in the total population, males and females. Females experienced the largest declines: DALYs -84.84% and ASDR -93.69%.

### Joinpoint regression analysis of smoking-attributable TB in China, 1990-2021

**Figure 1:**
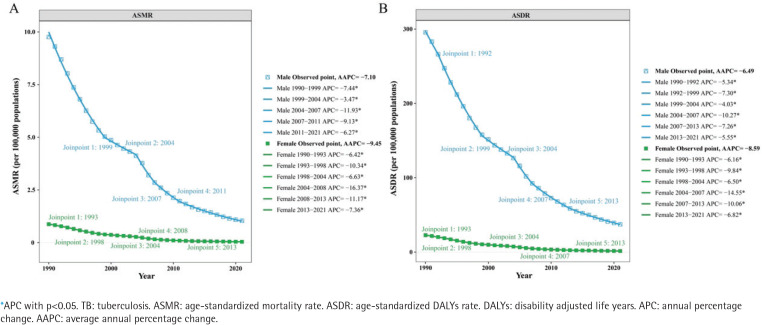
Joinpoint regression of ASMR and ASDR for smoking-attributable TB in Chinese males and females, 1990–2021: A) ASMR; B) ASDR. Blue represents males, green represents females

Joinpoint regression showed that both ASMR and ASDR declined steadily in Chinese males and females from 1990 to 2021. The AAPCs were -7.10% and -6.49% for males, and -9.45% and -8.59% for females, respectively (Figure 1, Supplementary file Table S1). For ASMR, the steepest decline in males occurred during 2004–2007 (APC= -11.93%; 95% CI: -14.26 – -9.54) and in females during 2004–2008 (APC= -16.37%; 95% CI: -17.62 – -15.10) (Figure 1A, Supplementary file Table S1). Likewise, ASDR fell most rapidly in 2004–2007 for both sexes: males -10.27% (95% CI: -11.81 – -8.70) and females -14.55% (95% CI: -16.54 – -12.51) (Figure 1B, Supplementary Table S1). Moreover, the EAPCs differed significantly between sexes for both ASMR and ASDR (p<0.001) (Supplementary file Table S2).

### Age-period-cohort effects on smoking-attributable TB mortality and DALY rates in China

**Figure 2:**
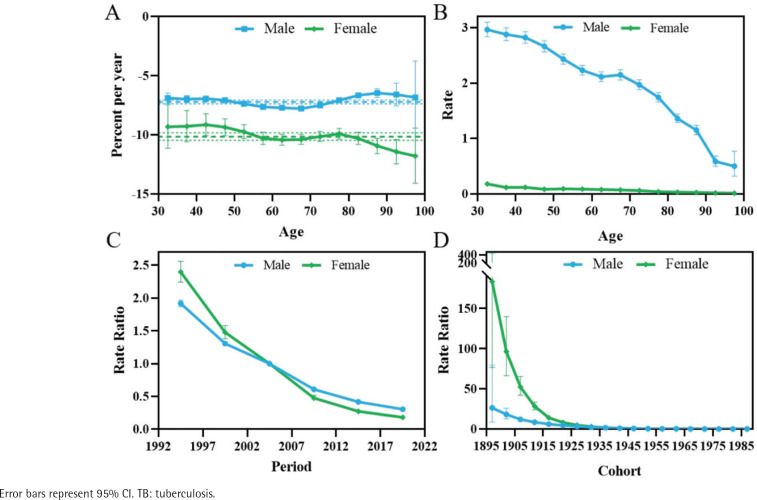
Age-period-cohort analysis of smoking-attributable TB mortality in Chinese males and females, 1990–2021: A) Net and local drift: temporal trends in smoking-attributable TB mortality 1990–2021; B) Age effect: fitted longitudinal age-specific mortality curves (per 100000), adjusted for period deviations; C) Period effect: relative risk of death for 1992–1996 to 2017–2021, expressed as age-specific rate ratios (reference period: 2002–2006); and D) Cohort effect: relative risk of death for birth cohorts 1895–1989, expressed as age-specific rate ratios (reference cohort: 1940–1944). Blue represents males, green represents females

Age-period-cohort modeling revealed a marked downward trend in smoking-attributable TB mortality from 1990 to 2021. Net drift was -7.21%/year (95% CI: -7.37 – -7.05) for males and -10.14%/year (95% CI: -10.46 – -9.82) for females. The largest local drift reductions were observed in males aged 65–69 years (-7.77%/year, 95% CI: -7.90 – -7.64) and females aged ≥95 years (-11.78%/year, 95% CI: -14.08 – -9.41) (Figure 2A, Supplementary file Table S3).

Age effects showed mortality peaking at 30–34 years for both males (2.96 per 100000) and females (0.18 per 100000), and declining thereafter. Males experienced a small rebound at 65–69 years (2.15 per 100000), whereas females declined steadily after the age of 50 years (Figure 2B, Supplementary file Table S4).

Period effects demonstrated a continuous fall in mortality risk for both males and females. RRs for males and females were 1.92 (95% CI: 1.87–1.97) and 2.40 (95% CI: 2.24–2.56) in 1992–1996, respectively, decreasing to 0.31 (95% CI: 0.29–0.32) and 0.18 (95% CI: 0.16–0.20) in 2017–2021 (Figure 2C, Supplementary file Table S5).

Cohort effects indicated declining mortality across successive birth cohorts for both males and females. Male cohort RRs fell from 26.33 (95% CI: 8.71–79.63) for 1895–1899 to 0.03 (95% CI: 0.03–0.04) for 1985–1989. Female cohort RRs dropped from 183.74 (95% CI: 76.63–440.57) to 0.01 (95% CI: 0.01–0.02) (Figure 2D, Supplementary file Table S6).

From 1990 to 2021, smoking-attributable TB DALY rates also declined markedly for both sexes. Net drift was -6.59%/year (95% CI: -6.90 – -6.28) for males and -9.30%/year (95% CI: -9.60 – -9.01) for females. The largest reductions occurred in males aged 55–59 years (-7.15%/year, 95% CI: -7.30 – -7.00) and females aged ≥95 years (-11.20%/year, 95% CI: -14.63 – -7.63) (Supplementary file: Figure S1A and Table S3). Age, period and cohort patterns for DALY rates closely mirrored those for mortality (Supplementary file: Figures S1B–S1D, Tables S3–S6).

### Trends in smoking-attributable TB burden 1990–2021 and BAPC projections to 2040

**Figure 3:**
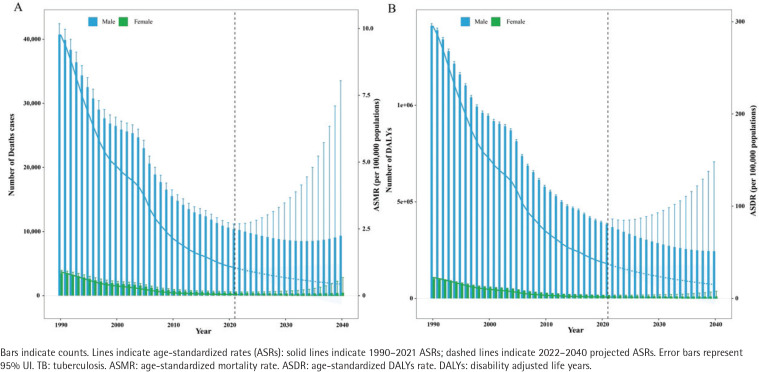
Temporal trends in smoking-attributable TB deaths and DALYs, and their ASRs in Chinese males and females, 1990–2040: A) Deaths; B) DALYs. Blue represents males, green represents females

Between 1990 and 2021, smoking-attributable TB deaths and ASMR in China declined continuously (Figure 3A). Male deaths fell from 40689 (95% UI: 28322–53781) to 10395 (95% UI: 7287–15404). Female deaths dropped from 3468 (95% UI: 2430–4777) to 451 (95% UI: 287–677) (Supplementary file Table S7). BAPC forecast indicates that deaths would initially decline and then rise again between 2022 and 2040, with turning points in 2034 for males (8509 deaths) and 2032 for females (345 deaths). By 2040, projected deaths were 9357 for males and 458 for females (Supplementary file Table S8). ASMR in males fell from 9.76 per 100000 (95 % UI: 6.86–12.95) in 1990 to 1.04 per 100000 (95 % UI: 0.74–1.55) in 2021, while in females it declined from 0.88 per 100000 (95 % UI: 0.60–1.21) to 0.04 per 100000 (95 % UI: 0.03–0.06). Projections to 2040 indicated further decreases to 0.43 per 100000 in males and 0.01 per 100000 in females (Supplementary file Tables S7 and S8). Male DALYs were projected to keep decreasing, whereas female DALYs would first decline and then increase after 2037 (turning point at 10012 DALYs). ASDR trends mirror those of ASMR (Figure 3B). The robustness of our projections was validated in the sensitivity analysis whose results were consistent with the above findings (Supplementary file Table S9).

### Decomposition of the smoking-attributable TB burden in China

The respective contributions of the three mutually exclusive drivers, population aging, population growth, and epidemiological changes, to the deaths and DALYs due to smoking-attributable TB in China were quantified next.

Between 1990 and 2021, deaths from smoking-attributable TB decreased by 75.44% in the total Chinese population aged ≥30 years. This net reduction resulted from the combined effects of a 21.39% increase attributable to population aging, a 54.62% increase from population growth, and a 151.45% decrease driven by epidemiological changes. These effects were most pronounced in females, where population aging and growth contributed to increases of 28.44% and 56.83%, respectively, while epidemiological changes led to a reduction of 172.27% (Supplementary file: Figure S2A and Table S10).

Similarly, the number of DALYs due to smoking-attributable TB in China decreased by 73.62% in the overall population. Population aging and population growth contributed increases of 12.19% and 53.28%, respectively, while epidemiological changes led to a substantial reduction of 139.08%. The magnitude of change in DALYs remained greatest in females (Supplementary file: Figure S2B and Table S10).

## DISCUSSION

Overall, the past three decades have witnessed a clear downward trend in deaths, DALYs, ASMR and ASDR attributable to smoking-related TB in China across the total population, males and females. However, the decline was markedly steeper among females than among males, suggesting that females may respond more sensitively to public health interventions or that the sex gap in smoking prevalence plays an important role. Age-period-cohort modeling further illuminated the underlying drivers. Age effects peaked at 30–34 years for both sexes and then declined. Nevertheless, male mortality showed a modest rebound at 65–69 years. Both period and cohort effects revealed sustained declines in mortality and DALY rates, with the 1985–1989 birth cohort significantly outperforming the 1895–1899 cohort. Although the overall disease burden was presently decreasing, BAPC projections indicated that deaths would rebound after 2022, with the inflection occurring in 2034 for males and 2032 for females. DALYs would continue to fall overall, yet among females a renewed increase was anticipated from 2037 onward. ASMR and the ASDR were expected to remain on a downward trajectory.

The number of deaths, DALYs, ASMR, and ASDR due to smoking-attributable TB in China all declined from 1990 to 2021. This trend aligns with the findings of a previous study^14^. Decomposition analysis indicated that epidemiological changes were the primary driver behind the reduction in the TB burden. This observed decline may be attributable to the continuous advancement of national TB control and prevention policies. Since the 1990s, TB has been designated a priority notifiable disease, accompanied by phased national control plans, gradual expansion of free anti-TB drugs, reinforced case-finding and reporting by primary health facilities^15,16^, and nationwide Directly Observed Therapy Shortcourse^17^. These measures significantly increased the detection rate of cases and the rate of standardized treatment, effectively reducing the spread of the disease and the occurrence of severe cases, laying the foundation for the decline in mortality rates and disease burden.

Marked sex disparities were evident: female ASMR and ASDR fell more sharply. There was far lower smoking prevalence among females^18^. The 2018 China Adult Tobacco Survey reported male smoking rates consistently above 50% and female smoking rates <5%^19^. Lower exposure and greater behavioral responsiveness to tobacco-control policies in females therefore translated into steeper declines in smoking-attributable TB. Second, broader sociocultural shifts have likely contributed positively to women’s health. Rising educational attainment and heightened sex-equality awareness have improved women’s employment opportunities, living standards, and sanitary conditions, while also fostering greater vigilance against secondhand smoke^20^. Shorter duration of passive smoke exposure has further reduced the risk of communicable diseases including TB. Third, women’s higher participation in health-education programs, combined with superior health literacy and medical adherence relative to men^21^, facilitates earlier diagnosis, standardized treatment, and better clinical outcomes. Previous studies indicate that women are more likely than men to seek care promptly and to follow medical advice^22^. The sex-specific pattern of smoking-attributable TB burden in China therefore reflects a complex interplay of these multifactorial determinants.

The age-period-cohort model results indicated that both male and female TB mortality and DALY rates peaked in the 30–34 years age group and then declined steadily. This suggests that smoking-attributable TB mortality was higher among young and middle-aged adults than among the elderly, a finding consistent with previous studies^23,24^. Smokers in this age bracket are often at the ‘peak’ of their smoking history: their daily cigarette consumption and smoking frequency are significantly higher than in older adults^25^, and a clear dose-response relationship exists between smoking intensity and both TB mortality and relapse risk. Compared with never smokers, current smokers have 1.7-fold to 2.9-fold higher mortality and 3.0-fold to 5.4-fold higher relapse risk^26^. We also observed a modest rebound in male TB mortality in the 65–69 years age group. As individuals age, pulmonary function and immune competence decline. Weaker immunity, compounded by malnutrition, pathogen exposure, and air pollution, renders older adults more susceptible to infection and disease. The cumulative effects of long-term tobacco exposure further amplify these risks^27,28^. Macro-level environmental shifts, historical background, economic policies, and social transitions, have generated distinct cohort effects among Chinese born in different eras. Our study shows that later-born cohorts face lower risks, echoing earlier findings^23^. First, China progressively established and refined its national TB control program from the mid-20th century onward, expanding early screening, standardized treatment, and, critically, universal BCG vaccination, which provided foundational protection for adolescents^16,29^. Second, rising public awareness of TB, socioeconomic development, improved nutrition, higher level of education, and better healthcare access have collectively strengthened resistance to TB and treatment adherence in younger generations^30,31^. Finally, China’s tobacco-control campaigns have improved risk perception among the young, who are more receptive to healthy lifestyles than earlier cohorts^32^, conferring an indirect protective effect against respiratory diseases such as TB.

Although ASMR and ASDR were projected to keep falling, the absolute numbers of TB deaths and DALYs may experience a ‘low-level rebound’ after an initial decline. Male deaths were expected to bottom out in 2034 and then rise. Female deaths will reach their nadir in 2032 before increasing. Male DALYs would continue to decrease, whereas female DALYs would increase after 2037. Multiple drivers underpin these trends. First, China is rapidly becoming a more aged society. The growing share of older adults, often burdened by comorbidities and waning immunity^33^, will heighten TB susceptibility and mortality risk^34^. Second, although tobacco-control policies have advanced, the tobacco-use landscape has become more complex^35^. While traditional smoking is declining, novel products such as e-cigarettes are gaining popularity among some young adults^36,37^, creating new health risks whose cumulative impact on TB epidemiology will emerge over time and may precipitate future rises in deaths and DALYs.

### Limitations

Our study has several limitations. First, the GBD data we used are derived from model-based integration of multiple surveillance sources. Although systematic bias-correction and modeling improved comparability and completeness, TB-related data for certain regions and age groups, especially the very old or remote populations, might still suffer from delayed reporting, under-ascertainment, or inconsistent quality. Second, the absence of individual-level data precluded adequate control for specific smoking patterns, occupational exposures, nutritional status and other potential confounders, leaving the results susceptible to residual confounding bias. Third, the primary analyses were conducted at the national population level, constituting an ecological design; the observed population-level statistical association between smoking and TB cannot be interpreted as individual-level causality. Fourth, BAPC projections assume that current trends will continue and may therefore fail to capture future shifts produced by policy interventions or environmental changes, limiting their practical utility. Fifth, the GBD 2021 database contains no information on e-cigarette use, yet the rapid uptake of vaping in China may already be altering the tobacco-exposure landscape. Sixth, we focused solely on smoking as a risk factor and did not incorporate interactions with air pollution, diabetes, HIV, or other comorbidities. Lastly, subgroup analyses on regions and examination of whether urban–rural disparities and regional inequalities may modify the association of smoking with TB were impossible since GBD 2021 provides no provincially stratified estimates, forcing the study to be conducted at the national level only.

## CONCLUSIONS

Smoking-attributable TB in China exhibits pronounced age- and sex-specific patterns. While age-standardized risks continue to decline, absolute deaths and DALYs may rise owing to population aging according to the projection to 2040. Therefore, while sustaining the decline in age-standardized risks, we must also monitor changes in the absolute numbers of deaths and DALYs. Smoking remains a critical and non-negligible risk factor for TB in China. To achieve the goals set by the National Tuberculosis Control Plan (2024–2030) and Healthy China 2030, it is imperative to continuously strengthen tobacco-control measures, maintain the favorable downward trajectory of TB burden, and guard against potential future increases.

## Supplementary Material



## Data Availability

Data sharing is not applicable to this article as no new data were created.
