# Supplementary material for: Tuberculosis burden attributable to smoking in China 1990–2021 and projections to 2040: A secondary analysis of GBD 2021 data
Source: Tob Induc Dis. 2026 Jul 20;24:10.18332/tid/215180. doi: 10.18332/tid/215180 (PMC13401253; doi:10.18332/tid/215180)
Supplement: Supplementary file 1 [file TID-24-116-s1.pdf]

Tobacco Induced Diseases

Supplementary file

© 2026 Zhao F. and Yan T.

DOI:
10.18332/tid/215180

The content has been provided by the author(s) and has not been reviewed, verified, or endorsed by European Publishing. It may not have undergone peer review. The views, opinions, and recommendations expressed are solely those of the author(s) and do not necessarily reflect the position of European Publishing. European Publishing accepts no responsibility or liability for any consequences arising from the use of, or reliance on, this content.

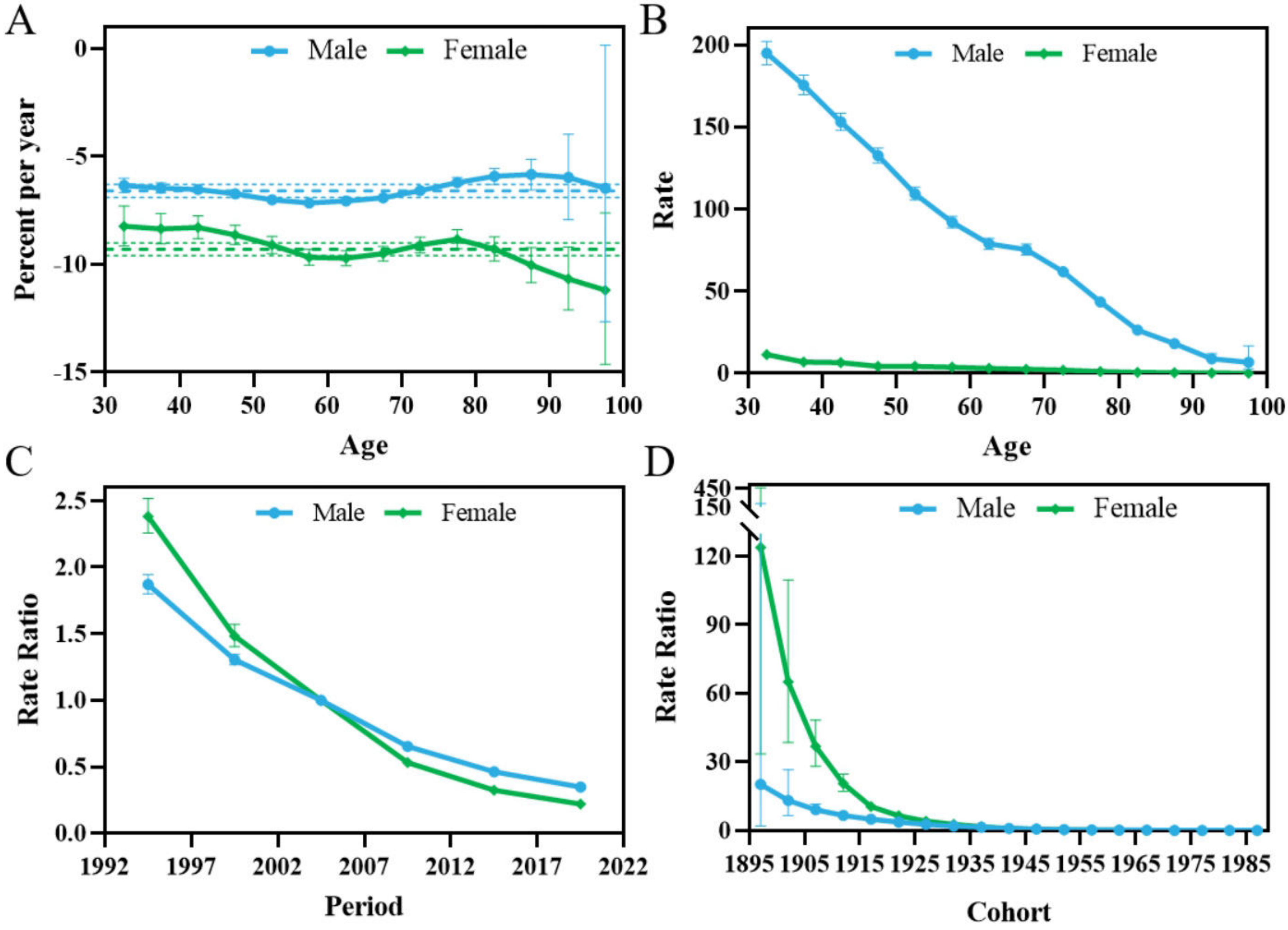
A
Male
Female
0
-5
-10
-15
Percent per year
30
40
50
60
70
80
90
100
Age
B
Male
Female
200
150
100
50
0
Rate
30
40
50
60
70
80
90
100
Age
C
Male
Female
2.5
2.0
1.5
1.0
0.5
0.0
Rate Ratio
1992
1997
2002
2007
2012
2017
2022
Period
D
Male
Female
450
150
120
90
60
30
0
Rate Ratio
1895
1905
1915
1925
1935
1945
1955
1965
1975
1985
Cohort

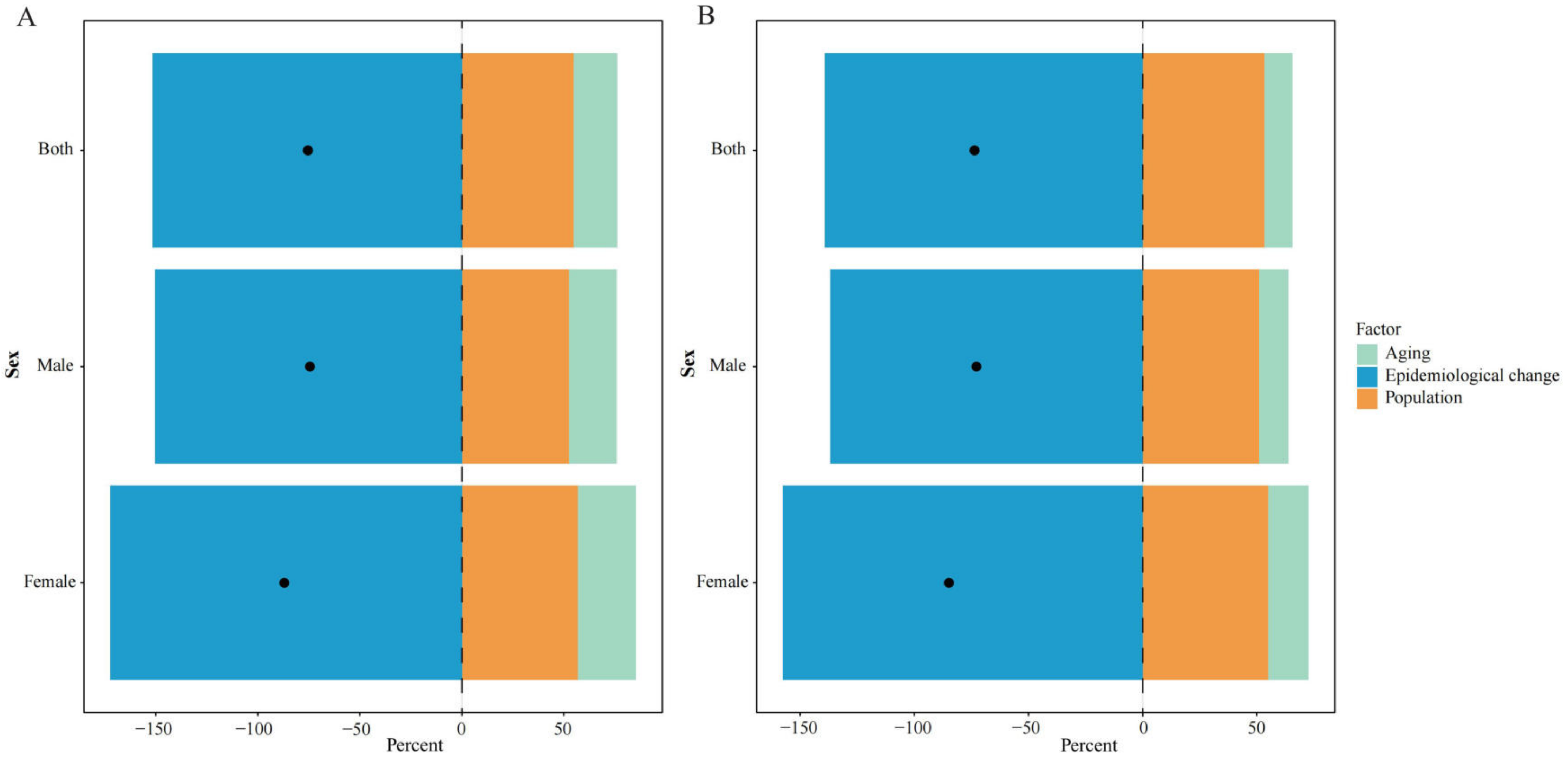

A
B
Both
Male
Female
Sex
−150
−100
−50
0
50
Percent
Factor
Aging
Epidemiological change
Population

S1. Joinpoint regression analysis of ASMR and ASDR due to smoking-attributable TB in Chi

measure	location	sex	cause	rei	age	joinpoint	AAPC.Index
Deaths	China	Male	Tuberculosis	Smoking	Age-standardized	4	Full Range
Deaths	China	Female	Tuberculosis	Smoking	Age-standardized	5	Full Range
DALYs	China	Male	Tuberculosis	Smoking	Age-standardized	5	Full Range
DALYs	China	Female	Tuberculosis	Smoking	Age-standardized	5	Full Range

measure	location	sex	cause	rei	age	joinpoint	Segment
Deaths	China	Male	Tuberculosis	Smoking	Age-standardized	4	0
Deaths	China	Male	Tuberculosis	Smoking	Age-standardized	4	1
Deaths	China	Male	Tuberculosis	Smoking	Age-standardized	4	2
Deaths	China	Male	Tuberculosis	Smoking	Age-standardized	4	3
Deaths	China	Male	Tuberculosis	Smoking	Age-standardized	4	4
Deaths	China	Female	Tuberculosis	Smoking	Age-standardized	5	0
Deaths	China	Female	Tuberculosis	Smoking	Age-standardized	5	1
Deaths	China	Female	Tuberculosis	Smoking	Age-standardized	5	2
Deaths	China	Female	Tuberculosis	Smoking	Age-standardized	5	3
Deaths	China	Female	Tuberculosis	Smoking	Age-standardized	5	4
Deaths	China	Female	Tuberculosis	Smoking	Age-standardized	5	5
DALYs	China	Male	Tuberculosis	Smoking	Age-standardized	5	0
DALYs	China	Male	Tuberculosis	Smoking	Age-standardized	5	1
DALYs	China	Male	Tuberculosis	Smoking	Age-standardized	5	2
DALYs	China	Male	Tuberculosis	Smoking	Age-standardized	5	3
DALYs	China	Male	Tuberculosis	Smoking	Age-standardized	5	4
DALYs	China	Male	Tuberculosis	Smoking	Age-standardized	5	5
DALYs	China	Female	Tuberculosis	Smoking	Age-standardized	5	0
DALYs	China	Female	Tuberculosis	Smoking	Age-standardized	5	1
DALYs	China	Female	Tuberculosis	Smoking	Age-standardized	5	2
DALYs	China	Female	Tuberculosis	Smoking	Age-standardized	5	3
DALYs	China	Female	Tuberculosis	Smoking	Age-standardized	5	4
DALYs	China	Female	Tuberculosis	Smoking	Age-standardized	5	5

TB, tuberculosis; ASMR, age-standardized mortality rate; ASDR, age-standardized disability adjus

nese males and females, 1990-2021.

Start.Obs	End.Obs	AAPC_95CI	Significant_indicator	Test.Statistic	P.Value
1990	2021	-7.10 (-7.42 to -6.78)	Yes	-41,57	0
1990	2021	-9.45 (-9.77 to -9.12)	Yes	-54,58	0
1990	2021	-6.49 (-6.73 to -6.25)	Yes	-50,91	0
1990	2021	-8.59 (-8.87 to -8.31)	Yes	-57,52	0

Segment.Start	Segment.End	APC_95CI	Significant_indicator	Test.Statistic	P.Value
1990	1999	-7.44 (-7.72 to -7.16)	Yes	-54,40	0
1999	2004	-3.47 (-4.39 to -2.55)	Yes	-7,80	0
2004	2007	-11.93 (-14.26 to -9.54)	Yes	-9,95	0
2007	2011	-9.13 (-10.36 to -7.87)	Yes	-14,69	0
2011	2021	-6.27 (-6.54 to -5.99)	Yes	-46,21	0
1990	1993	-6.42 (-8.05 to -4.77)	Yes	-8,09	0
1993	1998	-10.34 (-11.26 to -9.42)	Yes	-22,75	0
1998	2004	-6.63 (-7.27 to -5.99)	Yes	-21,26	0
2004	2008	-16.37 (-17.62 to -15.10)	Yes	-25,24	0
2008	2013	-11.17 (-12.05 to -10.27)	Yes	-25,10	0
2013	2021	-7.36 (-7.76 to -6.97)	Yes	-37,94	0
1990	1992	-5.34 (-7.58 to -3.05)	Yes	-4,89	0
1992	1999	-7.30 (-7.61 to -6.99)	Yes	-48,23	0
1999	2004	-4.03 (-4.61 to -3.44)	Yes	-14,26	0
2004	2007	-10.27 (-11.81 to -8.70)	Yes	-13,29	0
2007	2013	-7.26 (-7.64 to -6.87)	Yes	-38,76	0
2013	2021	-5.55 (-5.80 to -5.30)	Yes	-45,40	0
1990	1993	-6.16 (-7.40 to -4.90)	Yes	-10,18	0
1993	1998	-9.84 (-10.54 to -9.15)	Yes	-28,60	0
1998	2004	-6.50 (-6.98 to -6.02)	Yes	-27,90	0
2004	2007	-14.55 (-16.54 to -12.51)	Yes	-14,21	0
2007	2013	-10.06 (-10.53 to -9.59)	Yes	-43,19	0
2013	2021	-6.82 (-7.11 to -6.53)	Yes	-48,33	0

ted life year rate; AAPC, average annual percentage change; APC, annual percentage change; CI, confidence

S2. Comparison of the EAPCs in mortality rates and DALYs for smoking-attributable TB between ma

Measure	EAPC (95% CI) Male	EAPC (95% CI) Female	P-value
**Deaths**	-7.24 (-7.44, -7.04)	-10.11 (-10.43, -9.79)	<0.001
**DALYs**	-6.68 (-6.81, -6.54)	-9.10 (-9.33, -8.87)	<0.001

EAPC, estimated annual percentage change; DALYs, disability adjusted life years; TB, tuberculosis; CI, confidence interval.

S3. Net drift and local drift values for smoking-attributable TB mortality and D

measure	location	sex	cause	rei	Net Drift (%/year)	CI Low
Deaths	China	Male	Tuberculosis	Smoking	-7,21	-7,37
Deaths	China	Female	Tuberculosis	Smoking	-10,14	-10,46
DALYs	China	Male	Tuberculosis	Smoking	-6,59	-6,90
DALYs	China	Female	Tuberculosis	Smoking	-9,30	-9,60

measure	location	sex	cause	rei	label	Age
Deaths	China	Male	Tuberculosis	Smoking	30 to 34	32,5
Deaths	China	Male	Tuberculosis	Smoking	35 to 39	37,5
Deaths	China	Male	Tuberculosis	Smoking	40 to 44	42,5
Deaths	China	Male	Tuberculosis	Smoking	45 to 49	47,5
Deaths	China	Male	Tuberculosis	Smoking	50 to 54	52,5
Deaths	China	Male	Tuberculosis	Smoking	55 to 59	57,5
Deaths	China	Male	Tuberculosis	Smoking	60 to 64	62,5
Deaths	China	Male	Tuberculosis	Smoking	65 to 69	67,5
Deaths	China	Male	Tuberculosis	Smoking	70 to 74	72,5
Deaths	China	Male	Tuberculosis	Smoking	75 to 79	77,5
Deaths	China	Male	Tuberculosis	Smoking	80 to 84	82,5
Deaths	China	Male	Tuberculosis	Smoking	85 to 89	87,5
Deaths	China	Male	Tuberculosis	Smoking	90 to 94	92,5
Deaths	China	Male	Tuberculosis	Smoking	95 plus	97,5
Deaths	China	Female	Tuberculosis	Smoking	30 to 34	32,5
Deaths	China	Female	Tuberculosis	Smoking	35 to 39	37,5
Deaths	China	Female	Tuberculosis	Smoking	40 to 44	42,5
Deaths	China	Female	Tuberculosis	Smoking	45 to 49	47,5
Deaths	China	Female	Tuberculosis	Smoking	50 to 54	52,5
Deaths	China	Female	Tuberculosis	Smoking	55 to 59	57,5
Deaths	China	Female	Tuberculosis	Smoking	60 to 64	62,5
Deaths	China	Female	Tuberculosis	Smoking	65 to 69	67,5
Deaths	China	Female	Tuberculosis	Smoking	70 to 74	72,5
Deaths	China	Female	Tuberculosis	Smoking	75 to 79	77,5
Deaths	China	Female	Tuberculosis	Smoking	80 to 84	82,5
Deaths	China	Female	Tuberculosis	Smoking	85 to 89	87,5
Deaths	China	Female	Tuberculosis	Smoking	90 to 94	92,5
Deaths	China	Female	Tuberculosis	Smoking	95 plus	97,5
DALYs	China	Male	Tuberculosis	Smoking	30 to 34	32,5
DALYs	China	Male	Tuberculosis	Smoking	35 to 39	37,5
DALYs	China	Male	Tuberculosis	Smoking	40 to 44	42,5
DALYs	China	Male	Tuberculosis	Smoking	45 to 49	47,5
DALYs	China	Male	Tuberculosis	Smoking	50 to 54	52,5
DALYs	China	Male	Tuberculosis	Smoking	55 to 59	57,5
DALYs	China	Male	Tuberculosis	Smoking	60 to 64	62,5
DALYs	China	Male	Tuberculosis	Smoking	65 to 69	67,5
DALYs	China	Male	Tuberculosis	Smoking	70 to 74	72,5
DALYs	China	Male	Tuberculosis	Smoking	75 to 79	77,5
DALYs	China	Male	Tuberculosis	Smoking	80 to 84	82,5
DALYs	China	Male	Tuberculosis	Smoking	85 to 89	87,5
DALYs	China	Male	Tuberculosis	Smoking	90 to 94	92,5

S5. Period effects for smoking-attributable TB in China, represented by age-specific rate ratios of relative mortality and DALY risk for five-year periods from 1992-1996 to 2017-2021 (reference: 2002-2006).

measure	location	sex	cause	rei	label	Period	Rate Ratio	CI Lov	CI High
Deaths	China	Male	Tuberculosis	Smoking	1992 to 1996	1994,5	1,92	1,87	1,97
Deaths	China	Male	Tuberculosis	Smoking	1997 to 2001	1999,5	1,31	1,28	1,34
Deaths	China	Male	Tuberculosis	Smoking	2002 to 2006	2004,5	1,00	1,00	1,00
Deaths	China	Male	Tuberculosis	Smoking	2007 to 2011	2009,5	0,61	0,59	0,62
Deaths	China	Male	Tuberculosis	Smoking	2012 to 2016	2014,5	0,42	0,41	0,43
Deaths	China	Male	Tuberculosis	Smoking	2017 to 2021	2019,5	0,31	0,29	0,32
Deaths	China	Female	Tuberculosis	Smoking	1992 to 1996	1994,5	2,40	2,24	2,56
Deaths	China	Female	Tuberculosis	Smoking	1997 to 2001	1999,5	1,48	1,38	1,58
Deaths	China	Female	Tuberculosis	Smoking	2002 to 2006	2004,5	1,00	1,00	1,00
Deaths	China	Female	Tuberculosis	Smoking	2007 to 2011	2009,5	0,48	0,44	0,52
Deaths	China	Female	Tuberculosis	Smoking	2012 to 2016	2014,5	0,27	0,25	0,30
Deaths	China	Female	Tuberculosis	Smoking	2017 to 2021	2019,5	0,18	0,16	0,20
DALYs	China	Male	Tuberculosis	Smoking	1992 to 1996	1994,5	1,87	1,80	1,95
DALYs	China	Male	Tuberculosis	Smoking	1997 to 2001	1999,5	1,30	1,27	1,34
DALYs	China	Male	Tuberculosis	Smoking	2002 to 2006	2004,5	1,00	1,00	1,00
DALYs	China	Male	Tuberculosis	Smoking	2007 to 2011	2009,5	0,65	0,63	0,67
DALYs	China	Male	Tuberculosis	Smoking	2012 to 2016	2014,5	0,46	0,44	0,48
DALYs	China	Male	Tuberculosis	Smoking	2017 to 2021	2019,5	0,35	0,33	0,37
DALYs	China	Female	Tuberculosis	Smoking	1992 to 1996	1994,5	2,38	2,26	2,52
DALYs	China	Female	Tuberculosis	Smoking	1997 to 2001	1999,5	1,48	1,40	1,57
DALYs	China	Female	Tuberculosis	Smoking	2002 to 2006	2004,5	1,00	1,00	1,00
DALYs	China	Female	Tuberculosis	Smoking	2007 to 2011	2009,5	0,53	0,50	0,57
DALYs	China	Female	Tuberculosis	Smoking	2012 to 2016	2014,5	0,32	0,30	0,35
DALYs	China	Female	Tuberculosis	Smoking	2017 to 2021	2019,5	0,22	0,20	0,24

TB, tuberculosis; DALYs, disability adjusted life years; CI, confidence interval.

DALYs	China	Male	Tuberculosis	Smoking	95 plus	97,5
DALYs	China	Female	Tuberculosis	Smoking	30 to 34	32,5
DALYs	China	Female	Tuberculosis	Smoking	35 to 39	37,5
DALYs	China	Female	Tuberculosis	Smoking	40 to 44	42,5
DALYs	China	Female	Tuberculosis	Smoking	45 to 49	47,5
DALYs	China	Female	Tuberculosis	Smoking	50 to 54	52,5
DALYs	China	Female	Tuberculosis	Smoking	55 to 59	57,5
DALYs	China	Female	Tuberculosis	Smoking	60 to 64	62,5
DALYs	China	Female	Tuberculosis	Smoking	65 to 69	67,5
DALYs	China	Female	Tuberculosis	Smoking	70 to 74	72,5
DALYs	China	Female	Tuberculosis	Smoking	75 to 79	77,5
DALYs	China	Female	Tuberculosis	Smoking	80 to 84	82,5
DALYs	China	Female	Tuberculosis	Smoking	85 to 89	87,5
DALYs	China	Female	Tuberculosis	Smoking	90 to 94	92,5
DALYs	China	Female	Tuberculosis	Smoking	95 plus	97,5

TB, tuberculosis; DALYs, disability adjusted life years; CI, confidence interval.

ALY rates in China, 1990-2021.

CI High
-7,05
-9,82
-6,28
-9,01

Mean Percent Change per Calendar Year	CI Low	CI High
-6,89	-7,31	-6,46
-6,97	-7,27	-6,67
-6,93	-7,15	-6,71
-7,07	-7,24	-6,89
-7,36	-7,52	-7,21
-7,63	-7,78	-7,49
-7,71	-7,85	-7,58
-7,77	-7,90	-7,64
-7,50	-7,64	-7,37
-7,07	-7,23	-6,91
-6,66	-6,87	-6,44
-6,46	-6,83	-6,09
-6,59	-7,54	-5,62
-6,83	-9,82	-3,75
-9,31	-11,12	-7,46
-9,27	-10,57	-7,95
-9,13	-10,04	-8,20
-9,35	-10,05	-8,63
-9,73	-10,32	-9,13
-10,28	-10,80	-9,76
-10,41	-10,87	-9,95
-10,38	-10,80	-9,96
-10,13	-10,55	-9,71
-9,91	-10,36	-9,46
-10,30	-10,81	-9,79
-10,92	-11,57	-10,28
-11,42	-12,45	-10,38
-11,78	-14,08	-9,41
-6,34	-6,68	-6,01
-6,46	-6,70	-6,22
-6,52	-6,71	-6,33
-6,73	-6,89	-6,57
-7,00	-7,15	-6,85
-7,15	-7,30	-7,00
-7,06	-7,21	-6,91
-6,92	-7,07	-6,76
-6,58	-6,75	-6,40
-6,20	-6,43	-5,97
-5,92	-6,28	-5,55
-5,84	-6,54	-5,13
-5,97	-7,93	-3,97

-6,47	-12,67	0,16
-8,23	-9,14	-7,30
-8,35	-9,05	-7,65
-8,29	-8,81	-7,76
-8,63	-9,07	-8,19
-9,10	-9,50	-8,70
-9,67	-10,03	-9,30
-9,72	-10,06	-9,37
-9,51	-9,84	-9,17
-9,11	-9,47	-8,74
-8,84	-9,27	-8,40
-9,28	-9,84	-8,72
-10,03	-10,83	-9,23
-10,67	-12,11	-9,20
-11,20	-14,63	-7,63

S4. Age effects on smoking-attributable TB mortality ar

measure	location	sex	cause	rei	Age	label	Rate	CI Low	CI High
Deaths	China	Male	Tuberculosis	Smoking	32,5	30 to 34	2,96	2,84	3,10
Deaths	China	Male	Tuberculosis	Smoking	37,5	35 to 39	2,88	2,77	3,00
Deaths	China	Male	Tuberculosis	Smoking	42,5	40 to 44	2,82	2,72	2,93
Deaths	China	Male	Tuberculosis	Smoking	47,5	45 to 49	2,66	2,57	2,76
Deaths	China	Male	Tuberculosis	Smoking	52,5	50 to 54	2,43	2,34	2,53
Deaths	China	Male	Tuberculosis	Smoking	57,5	55 to 59	2,23	2,14	2,32
Deaths	China	Male	Tuberculosis	Smoking	62,5	60 to 64	2,11	2,03	2,20
Deaths	China	Male	Tuberculosis	Smoking	67,5	65 to 69	2,15	2,06	2,24
Deaths	China	Male	Tuberculosis	Smoking	72,5	70 to 74	1,97	1,88	2,06
Deaths	China	Male	Tuberculosis	Smoking	77,5	75 to 79	1,74	1,66	1,83
Deaths	China	Male	Tuberculosis	Smoking	82,5	80 to 84	1,36	1,29	1,44
Deaths	China	Male	Tuberculosis	Smoking	87,5	85 to 89	1,15	1,07	1,24
Deaths	China	Male	Tuberculosis	Smoking	92,5	90 to 94	0,58	0,50	0,69
Deaths	China	Male	Tuberculosis	Smoking	97,5	95 plus	0,50	0,32	0,77
Deaths	China	Female	Tuberculosis	Smoking	32,5	30 to 34	0,18	0,15	0,21
Deaths	China	Female	Tuberculosis	Smoking	37,5	35 to 39	0,12	0,10	0,14
Deaths	China	Female	Tuberculosis	Smoking	42,5	40 to 44	0,12	0,10	0,14
Deaths	China	Female	Tuberculosis	Smoking	47,5	45 to 49	0,08	0,07	0,10
Deaths	China	Female	Tuberculosis	Smoking	52,5	50 to 54	0,09	0,08	0,11
Deaths	China	Female	Tuberculosis	Smoking	57,5	55 to 59	0,09	0,08	0,10
Deaths	China	Female	Tuberculosis	Smoking	62,5	60 to 64	0,08	0,07	0,09
Deaths	China	Female	Tuberculosis	Smoking	67,5	65 to 69	0,07	0,06	0,08
Deaths	China	Female	Tuberculosis	Smoking	72,5	70 to 74	0,06	0,05	0,07
Deaths	China	Female	Tuberculosis	Smoking	77,5	75 to 79	0,04	0,03	0,05
Deaths	China	Female	Tuberculosis	Smoking	82,5	80 to 84	0,03	0,02	0,04
Deaths	China	Female	Tuberculosis	Smoking	87,5	85 to 89	0,02	0,02	0,03
Deaths	China	Female	Tuberculosis	Smoking	92,5	90 to 94	0,02	0,01	0,02
Deaths	China	Female	Tuberculosis	Smoking	97,5	95 plus	0,01	0,01	0,02
DALYs	China	Male	Tuberculosis	Smoking	32,5	30 to 34	194,83	187,84	202,08
DALYs	China	Male	Tuberculosis	Smoking	37,5	35 to 39	175,46	169,54	181,59
DALYs	China	Male	Tuberculosis	Smoking	42,5	40 to 44	153,08	148,02	158,31
DALYs	China	Male	Tuberculosis	Smoking	47,5	45 to 49	132,53	128,04	137,18
DALYs	China	Male	Tuberculosis	Smoking	52,5	50 to 54	109,32	105,45	113,35
DALYs	China	Male	Tuberculosis	Smoking	57,5	55 to 59	91,88	88,41	95,49
DALYs	China	Male	Tuberculosis	Smoking	62,5	60 to 64	78,77	75,52	82,16
DALYs	China	Male	Tuberculosis	Smoking	67,5	65 to 69	75,33	72,09	78,72
DALYs	China	Male	Tuberculosis	Smoking	72,5	70 to 74	61,80	58,90	64,85
DALYs	China	Male	Tuberculosis	Smoking	77,5	75 to 79	43,57	41,20	46,06
DALYs	China	Male	Tuberculosis	Smoking	82,5	80 to 84	26,42	24,55	28,44
DALYs	China	Male	Tuberculosis	Smoking	87,5	85 to 89	18,04	16,02	20,31
DALYs	China	Male	Tuberculosis	Smoking	92,5	90 to 94	8,85	6,52	12,01
DALYs	China	Male	Tuberculosis	Smoking	97,5	95 plus	6,78	2,76	16,66
DALYs	China	Female	Tuberculosis	Smoking	32,5	30 to 34	11,41	10,42	12,50
DALYs	China	Female	Tuberculosis	Smoking	37,5	35 to 39	6,96	6,34	7,65
DALYs	China	Female	Tuberculosis	Smoking	42,5	40 to 44	6,64	6,08	7,25
DALYs	China	Female	Tuberculosis	Smoking	47,5	45 to 49	4,34	3,94	4,78
DALYs	China	Female	Tuberculosis	Smoking	52,5	50 to 54	4,27	3,88	4,70
DALYs	China	Female	Tuberculosis	Smoking	57,5	55 to 59	3,75	3,39	4,14

DALYs	China	Female	Tuberculosis	Smoking	62,5	60 to 64	3,02	2,72	3,37
DALYs	China	Female	Tuberculosis	Smoking	67,5	65 to 69	2,51	2,25	2,80
DALYs	China	Female	Tuberculosis	Smoking	72,5	70 to 74	1,96	1,74	2,20
DALYs	China	Female	Tuberculosis	Smoking	77,5	75 to 79	1,05	0,92	1,20
DALYs	China	Female	Tuberculosis	Smoking	82,5	80 to 84	0,63	0,54	0,74
DALYs	China	Female	Tuberculosis	Smoking	87,5	85 to 89	0,43	0,35	0,52
DALYs	China	Female	Tuberculosis	Smoking	92,5	90 to 94	0,26	0,19	0,35
DALYs	China	Female	Tuberculosis	Smoking	97,5	95 plus	0,17	0,09	0,33

TB, tuberculosis; DALYs, disability adjusted life years; CI, confidence interval.

S6. Cohort effects for smoking-attributable TB in China, represented by age-specific rate ratios of relative mortality and DALY risk for birth cohorts from 1895 to 1989 (reference: 1940-1944).

measure	location	sex	cause	rei	label	Cohort	Rate Ratio	CI Lo	CI High
Deaths	China	Male	Tuberculosis	Smoking	1895 to 1899	1897	26,33	8,71	79,63
Deaths	China	Male	Tuberculosis	Smoking	1900 to 1904	1902	18,35	12,99	25,92
Deaths	China	Male	Tuberculosis	Smoking	1905 to 1909	1907	12,09	10,66	13,72
Deaths	China	Male	Tuberculosis	Smoking	1910 to 1914	1912	8,47	7,87	9,10
Deaths	China	Male	Tuberculosis	Smoking	1915 to 1919	1917	6,24	5,93	6,56
Deaths	China	Male	Tuberculosis	Smoking	1920 to 1924	1922	4,54	4,36	4,72
Deaths	China	Male	Tuberculosis	Smoking	1925 to 1929	1927	3,24	3,13	3,35
Deaths	China	Male	Tuberculosis	Smoking	1930 to 1934	1932	2,22	2,15	2,29
Deaths	China	Male	Tuberculosis	Smoking	1935 to 1939	1937	1,51	1,47	1,56
Deaths	China	Male	Tuberculosis	Smoking	1940 to 1944	1942	1,00	1,00	1,00
Deaths	China	Male	Tuberculosis	Smoking	1945 to 1949	1947	0,65	0,63	0,67
Deaths	China	Male	Tuberculosis	Smoking	1950 to 1954	1952	0,43	0,42	0,45
Deaths	China	Male	Tuberculosis	Smoking	1955 to 1959	1957	0,31	0,30	0,32
Deaths	China	Male	Tuberculosis	Smoking	1960 to 1964	1962	0,21	0,20	0,21
Deaths	China	Male	Tuberculosis	Smoking	1965 to 1969	1967	0,15	0,14	0,15
Deaths	China	Male	Tuberculosis	Smoking	1970 to 1974	1972	0,10	0,10	0,11
Deaths	China	Male	Tuberculosis	Smoking	1975 to 1979	1977	0,07	0,07	0,08
Deaths	China	Male	Tuberculosis	Smoking	1980 to 1984	1982	0,05	0,04	0,05
Deaths	China	Male	Tuberculosis	Smoking	1985 to 1989	1987	0,03	0,03	0,04
Deaths	China	Female	Tuberculosis	Smoking	1895 to 1899	1897	183,74	76,63	440,57
Deaths	China	Female	Tuberculosis	Smoking	1900 to 1904	1902	96,38	66,37	139,95
Deaths	China	Female	Tuberculosis	Smoking	1905 to 1909	1907	52,47	42,08	65,44
Deaths	China	Female	Tuberculosis	Smoking	1910 to 1914	1912	28,36	23,96	33,56
Deaths	China	Female	Tuberculosis	Smoking	1915 to 1919	1917	13,99	12,16	16,10
Deaths	China	Female	Tuberculosis	Smoking	1920 to 1924	1922	8,23	7,30	9,28
Deaths	China	Female	Tuberculosis	Smoking	1925 to 1929	1927	4,84	4,34	5,39
Deaths	China	Female	Tuberculosis	Smoking	1930 to 1934	1932	2,94	2,66	3,25
Deaths	China	Female	Tuberculosis	Smoking	1935 to 1939	1937	1,79	1,62	1,98
Deaths	China	Female	Tuberculosis	Smoking	1940 to 1944	1942	1,00	1,00	1,00
Deaths	China	Female	Tuberculosis	Smoking	1945 to 1949	1947	0,56	0,50	0,62
Deaths	China	Female	Tuberculosis	Smoking	1950 to 1954	1952	0,32	0,28	0,36
Deaths	China	Female	Tuberculosis	Smoking	1955 to 1959	1957	0,20	0,17	0,23
Deaths	China	Female	Tuberculosis	Smoking	1960 to 1964	1962	0,12	0,10	0,14
Deaths	China	Female	Tuberculosis	Smoking	1965 to 1969	1967	0,08	0,06	0,09
Deaths	China	Female	Tuberculosis	Smoking	1970 to 1974	1972	0,05	0,04	0,06
Deaths	China	Female	Tuberculosis	Smoking	1975 to 1979	1977	0,03	0,02	0,04
Deaths	China	Female	Tuberculosis	Smoking	1980 to 1984	1982	0,02	0,01	0,03
Deaths	China	Female	Tuberculosis	Smoking	1985 to 1989	1987	0,01	0,01	0,02
DALYs	China	Male	Tuberculosis	Smoking	1895 to 1899	1897	20,23	1,96	208,82
DALYs	China	Male	Tuberculosis	Smoking	1900 to 1904	1902	13,11	6,44	26,67
DALYs	China	Male	Tuberculosis	Smoking	1905 to 1909	1907	9,17	7,19	11,69
DALYs	China	Male	Tuberculosis	Smoking	1910 to 1914	1912	6,61	5,85	7,48
DALYs	China	Male	Tuberculosis	Smoking	1915 to 1919	1917	4,97	4,62	5,35
DALYs	China	Male	Tuberculosis	Smoking	1920 to 1924	1922	3,72	3,52	3,92
DALYs	China	Male	Tuberculosis	Smoking	1925 to 1929	1927	2,77	2,65	2,89
DALYs	China	Male	Tuberculosis	Smoking	1930 to 1934	1932	2,00	1,92	2,07

DALYs	China	Male	Tuberculosis	Smoking	1935 to 1939	1937	1,43	1,38	1,49
DALYs	China	Male	Tuberculosis	Smoking	1940 to 1944	1942	1,00	1,00	1,00
DALYs	China	Male	Tuberculosis	Smoking	1945 to 1949	1947	0,68	0,65	0,70
DALYs	China	Male	Tuberculosis	Smoking	1950 to 1954	1952	0,46	0,45	0,48
DALYs	China	Male	Tuberculosis	Smoking	1955 to 1959	1957	0,33	0,32	0,34
DALYs	China	Male	Tuberculosis	Smoking	1960 to 1964	1962	0,23	0,22	0,23
DALYs	China	Male	Tuberculosis	Smoking	1965 to 1969	1967	0,16	0,16	0,17
DALYs	China	Male	Tuberculosis	Smoking	1970 to 1974	1972	0,12	0,11	0,13
DALYs	China	Male	Tuberculosis	Smoking	1975 to 1979	1977	0,09	0,08	0,09
DALYs	China	Male	Tuberculosis	Smoking	1980 to 1984	1982	0,06	0,06	0,07
DALYs	China	Male	Tuberculosis	Smoking	1985 to 1989	1987	0,04	0,04	0,05
DALYs	China	Female	Tuberculosis	Smoking	1895 to 1899	1897	123,88	33,58	457,04
DALYs	China	Female	Tuberculosis	Smoking	1900 to 1904	1902	65,12	38,67	109,68
DALYs	China	Female	Tuberculosis	Smoking	1905 to 1909	1907	36,88	28,15	48,31
DALYs	China	Female	Tuberculosis	Smoking	1910 to 1914	1912	20,60	17,17	24,72
DALYs	China	Female	Tuberculosis	Smoking	1915 to 1919	1917	10,46	9,11	12,01
DALYs	China	Female	Tuberculosis	Smoking	1920 to 1924	1922	6,53	5,87	7,27
DALYs	China	Female	Tuberculosis	Smoking	1925 to 1929	1927	4,06	3,71	4,45
DALYs	China	Female	Tuberculosis	Smoking	1930 to 1934	1932	2,65	2,44	2,88
DALYs	China	Female	Tuberculosis	Smoking	1935 to 1939	1937	1,70	1,58	1,84
DALYs	China	Female	Tuberculosis	Smoking	1940 to 1944	1942	1,00	1,00	1,00
DALYs	China	Female	Tuberculosis	Smoking	1945 to 1949	1947	0,59	0,54	0,64
DALYs	China	Female	Tuberculosis	Smoking	1950 to 1954	1952	0,34	0,31	0,37
DALYs	China	Female	Tuberculosis	Smoking	1955 to 1959	1957	0,22	0,20	0,24
DALYs	China	Female	Tuberculosis	Smoking	1960 to 1964	1962	0,14	0,12	0,15
DALYs	China	Female	Tuberculosis	Smoking	1965 to 1969	1967	0,09	0,08	0,11
DALYs	China	Female	Tuberculosis	Smoking	1970 to 1974	1972	0,06	0,05	0,07
DALYs	China	Female	Tuberculosis	Smoking	1975 to 1979	1977	0,04	0,03	0,05
DALYs	China	Female	Tuberculosis	Smoking	1980 to 1984	1982	0,02	0,02	0,03
DALYs	China	Female	Tuberculosis	Smoking	1985 to 1989	1987	0,02	0,01	0,02

DALYs, disability adjusted life years; TB, tuberculosis; CI, confidence interval.

S7. Numbers of deaths and DALYs and their ASRs due to smoking-attributable TB in China, 1990-2021.

measure	location	sex	age	cause	rei	metric	year	val	lower	upper
Deaths	China	Male	All ages	Tuberculosis	Smoking	Number	1990	40689	28322	53781
Deaths	China	Male	All ages	Tuberculosis	Smoking	Number	1991	39932	29541	51641
Deaths	China	Male	All ages	Tuberculosis	Smoking	Number	1992	38374	28287	51551
Deaths	China	Male	All ages	Tuberculosis	Smoking	Number	1993	36408	28024	47202
Deaths	China	Male	All ages	Tuberculosis	Smoking	Number	1994	34325	26538	44304
Deaths	China	Male	All ages	Tuberculosis	Smoking	Number	1995	32545	25631	40951
Deaths	China	Male	All ages	Tuberculosis	Smoking	Number	1996	30792	23853	38194
Deaths	China	Male	All ages	Tuberculosis	Smoking	Number	1997	29003	22791	37113
Deaths	China	Male	All ages	Tuberculosis	Smoking	Number	1998	27633	21955	34975
Deaths	China	Male	All ages	Tuberculosis	Smoking	Number	1999	26813	21086	33209
Deaths	China	Male	All ages	Tuberculosis	Smoking	Number	2000	26557	20508	33337
Deaths	China	Male	All ages	Tuberculosis	Smoking	Number	2001	25873	20343	32923
Deaths	China	Male	All ages	Tuberculosis	Smoking	Number	2002	25569	19874	32113
Deaths	China	Male	All ages	Tuberculosis	Smoking	Number	2003	25343	19349	32232
Deaths	China	Male	All ages	Tuberculosis	Smoking	Number	2004	24791	18963	30891
Deaths	China	Male	All ages	Tuberculosis	Smoking	Number	2005	23132	18482	28705
Deaths	China	Male	All ages	Tuberculosis	Smoking	Number	2006	20461	16114	24960
Deaths	China	Male	All ages	Tuberculosis	Smoking	Number	2007	18832	14714	22862
Deaths	China	Male	All ages	Tuberculosis	Smoking	Number	2008	17785	14119	21731
Deaths	China	Male	All ages	Tuberculosis	Smoking	Number	2009	16543	13169	20220
Deaths	China	Male	All ages	Tuberculosis	Smoking	Number	2010	15485	11967	19324
Deaths	China	Male	All ages	Tuberculosis	Smoking	Number	2011	14795	11634	18604
Deaths	China	Male	All ages	Tuberculosis	Smoking	Number	2012	14222	10906	18130
Deaths	China	Male	All ages	Tuberculosis	Smoking	Number	2013	13473	10214	16759
Deaths	China	Male	All ages	Tuberculosis	Smoking	Number	2014	12930	9795	16617
Deaths	China	Male	All ages	Tuberculosis	Smoking	Number	2015	12713	9618	16367
Deaths	China	Male	All ages	Tuberculosis	Smoking	Number	2016	12455	9182	16444
Deaths	China	Male	All ages	Tuberculosis	Smoking	Number	2017	11849	8801	16149
Deaths	China	Male	All ages	Tuberculosis	Smoking	Number	2018	11317	7986	15225
Deaths	China	Male	All ages	Tuberculosis	Smoking	Number	2019	10881	7385	15555
Deaths	China	Male	All ages	Tuberculosis	Smoking	Number	2020	10603	7278	15651

Deaths	China	Male	All ages	Tuberculosis	Smoking	Number	2021	10395	7287	15404
Deaths	China	Male	Age-standardized	Tuberculosis	Smoking	Rate	1990	9,76	6,86	12,95
Deaths	China	Male	Age-standardized	Tuberculosis	Smoking	Rate	1991	9,31	6,78	12,03
Deaths	China	Male	Age-standardized	Tuberculosis	Smoking	Rate	1992	8,70	6,41	11,77
Deaths	China	Male	Age-standardized	Tuberculosis	Smoking	Rate	1993	8,03	6,14	10,39
Deaths	China	Male	Age-standardized	Tuberculosis	Smoking	Rate	1994	7,37	5,71	9,51
Deaths	China	Male	Age-standardized	Tuberculosis	Smoking	Rate	1995	6,80	5,31	8,64
Deaths	China	Male	Age-standardized	Tuberculosis	Smoking	Rate	1996	6,26	4,86	7,88
Deaths	China	Male	Age-standardized	Tuberculosis	Smoking	Rate	1997	5,74	4,50	7,31
Deaths	China	Male	Age-standardized	Tuberculosis	Smoking	Rate	1998	5,33	4,20	6,77
Deaths	China	Male	Age-standardized	Tuberculosis	Smoking	Rate	1999	5,03	3,94	6,24
Deaths	China	Male	Age-standardized	Tuberculosis	Smoking	Rate	2000	4,86	3,73	6,12
Deaths	China	Male	Age-standardized	Tuberculosis	Smoking	Rate	2001	4,63	3,63	5,94
Deaths	China	Male	Age-standardized	Tuberculosis	Smoking	Rate	2002	4,46	3,47	5,61
Deaths	China	Male	Age-standardized	Tuberculosis	Smoking	Rate	2003	4,32	3,31	5,51
Deaths	China	Male	Age-standardized	Tuberculosis	Smoking	Rate	2004	4,13	3,17	5,17
Deaths	China	Male	Age-standardized	Tuberculosis	Smoking	Rate	2005	3,77	3,01	4,69
Deaths	China	Male	Age-standardized	Tuberculosis	Smoking	Rate	2006	3,20	2,51	3,92
Deaths	China	Male	Age-standardized	Tuberculosis	Smoking	Rate	2007	2,85	2,23	3,50
Deaths	China	Male	Age-standardized	Tuberculosis	Smoking	Rate	2008	2,60	2,06	3,19
Deaths	China	Male	Age-standardized	Tuberculosis	Smoking	Rate	2009	2,35	1,86	2,89
Deaths	China	Male	Age-standardized	Tuberculosis	Smoking	Rate	2010	2,13	1,64	2,65
Deaths	China	Male	Age-standardized	Tuberculosis	Smoking	Rate	2011	1,97	1,55	2,48
Deaths	China	Male	Age-standardized	Tuberculosis	Smoking	Rate	2012	1,84	1,42	2,34
Deaths	China	Male	Age-standardized	Tuberculosis	Smoking	Rate	2013	1,69	1,29	2,10
Deaths	China	Male	Age-standardized	Tuberculosis	Smoking	Rate	2014	1,57	1,20	2,02
Deaths	China	Male	Age-standardized	Tuberculosis	Smoking	Rate	2015	1,50	1,15	1,93
Deaths	China	Male	Age-standardized	Tuberculosis	Smoking	Rate	2016	1,43	1,05	1,89
Deaths	China	Male	Age-standardized	Tuberculosis	Smoking	Rate	2017	1,32	0,98	1,80
Deaths	China	Male	Age-standardized	Tuberculosis	Smoking	Rate	2018	1,23	0,87	1,64
Deaths	China	Male	Age-standardized	Tuberculosis	Smoking	Rate	2019	1,15	0,79	1,63
Deaths	China	Male	Age-standardized	Tuberculosis	Smoking	Rate	2020	1,09	0,75	1,60
Deaths	China	Male	Age-standardized	Tuberculosis	Smoking	Rate	2021	1,04	0,74	1,55

Deaths	China	Female	All ages	Tuberculosis	Smoking	Number	1990	3468	2430	4777
Deaths	China	Female	All ages	Tuberculosis	Smoking	Number	1991	3379	2403	4625
Deaths	China	Female	All ages	Tuberculosis	Smoking	Number	1992	3241	2330	4462
Deaths	China	Female	All ages	Tuberculosis	Smoking	Number	1993	3057	2191	4074
Deaths	China	Female	All ages	Tuberculosis	Smoking	Number	1994	2841	2014	3777
Deaths	China	Female	All ages	Tuberculosis	Smoking	Number	1995	2616	1865	3533
Deaths	China	Female	All ages	Tuberculosis	Smoking	Number	1996	2414	1755	3294
Deaths	China	Female	All ages	Tuberculosis	Smoking	Number	1997	2210	1617	2930
Deaths	China	Female	All ages	Tuberculosis	Smoking	Number	1998	2048	1498	2691
Deaths	China	Female	All ages	Tuberculosis	Smoking	Number	1999	1950	1419	2568
Deaths	China	Female	All ages	Tuberculosis	Smoking	Number	2000	1911	1383	2559
Deaths	China	Female	All ages	Tuberculosis	Smoking	Number	2001	1822	1323	2393
Deaths	China	Female	All ages	Tuberculosis	Smoking	Number	2002	1777	1311	2320
Deaths	China	Female	All ages	Tuberculosis	Smoking	Number	2003	1726	1249	2223
Deaths	China	Female	All ages	Tuberculosis	Smoking	Number	2004	1619	1199	2113
Deaths	China	Female	All ages	Tuberculosis	Smoking	Number	2005	1435	1042	1902
Deaths	China	Female	All ages	Tuberculosis	Smoking	Number	2006	1218	912	1608
Deaths	China	Female	All ages	Tuberculosis	Smoking	Number	2007	1063	783	1372
Deaths	China	Female	All ages	Tuberculosis	Smoking	Number	2008	952	696	1230
Deaths	China	Female	All ages	Tuberculosis	Smoking	Number	2009	858	619	1134
Deaths	China	Female	All ages	Tuberculosis	Smoking	Number	2010	780	582	1022
Deaths	China	Female	All ages	Tuberculosis	Smoking	Number	2011	723	518	956
Deaths	China	Female	All ages	Tuberculosis	Smoking	Number	2012	673	480	892
Deaths	China	Female	All ages	Tuberculosis	Smoking	Number	2013	619	440	836
Deaths	China	Female	All ages	Tuberculosis	Smoking	Number	2014	579	409	786
Deaths	China	Female	All ages	Tuberculosis	Smoking	Number	2015	567	386	777
Deaths	China	Female	All ages	Tuberculosis	Smoking	Number	2016	558	371	761
Deaths	China	Female	All ages	Tuberculosis	Smoking	Number	2017	524	360	726
Deaths	China	Female	All ages	Tuberculosis	Smoking	Number	2018	496	332	700
Deaths	China	Female	All ages	Tuberculosis	Smoking	Number	2019	474	314	686
Deaths	China	Female	All ages	Tuberculosis	Smoking	Number	2020	461	299	681
Deaths	China	Female	All ages	Tuberculosis	Smoking	Number	2021	451	287	677
Deaths	China	Female	Age-standardized	Tuberculosis	Smoking	Rate	1990	0,88	0,60	1,21

Deaths	China	Female	Age-standardized	Tuberculosis	Smoking	Rate	1991	0,83	0,59	1,13
Deaths	China	Female	Age-standardized	Tuberculosis	Smoking	Rate	1992	0,78	0,56	1,05
Deaths	China	Female	Age-standardized	Tuberculosis	Smoking	Rate	1993	0,72	0,51	0,95
Deaths	China	Female	Age-standardized	Tuberculosis	Smoking	Rate	1994	0,65	0,46	0,87
Deaths	China	Female	Age-standardized	Tuberculosis	Smoking	Rate	1995	0,58	0,41	0,79
Deaths	China	Female	Age-standardized	Tuberculosis	Smoking	Rate	1996	0,52	0,37	0,71
Deaths	China	Female	Age-standardized	Tuberculosis	Smoking	Rate	1997	0,47	0,34	0,62
Deaths	China	Female	Age-standardized	Tuberculosis	Smoking	Rate	1998	0,42	0,30	0,55
Deaths	China	Female	Age-standardized	Tuberculosis	Smoking	Rate	1999	0,39	0,28	0,51
Deaths	China	Female	Age-standardized	Tuberculosis	Smoking	Rate	2000	0,37	0,26	0,49
Deaths	China	Female	Age-standardized	Tuberculosis	Smoking	Rate	2001	0,34	0,25	0,45
Deaths	China	Female	Age-standardized	Tuberculosis	Smoking	Rate	2002	0,32	0,24	0,42
Deaths	China	Female	Age-standardized	Tuberculosis	Smoking	Rate	2003	0,30	0,22	0,39
Deaths	China	Female	Age-standardized	Tuberculosis	Smoking	Rate	2004	0,27	0,20	0,36
Deaths	China	Female	Age-standardized	Tuberculosis	Smoking	Rate	2005	0,24	0,17	0,31
Deaths	China	Female	Age-standardized	Tuberculosis	Smoking	Rate	2006	0,19	0,14	0,25
Deaths	China	Female	Age-standardized	Tuberculosis	Smoking	Rate	2007	0,16	0,12	0,21
Deaths	China	Female	Age-standardized	Tuberculosis	Smoking	Rate	2008	0,14	0,10	0,18
Deaths	China	Female	Age-standardized	Tuberculosis	Smoking	Rate	2009	0,12	0,09	0,16
Deaths	China	Female	Age-standardized	Tuberculosis	Smoking	Rate	2010	0,11	0,08	0,14
Deaths	China	Female	Age-standardized	Tuberculosis	Smoking	Rate	2011	0,09	0,07	0,13
Deaths	China	Female	Age-standardized	Tuberculosis	Smoking	Rate	2012	0,09	0,06	0,11
Deaths	China	Female	Age-standardized	Tuberculosis	Smoking	Rate	2013	0,08	0,05	0,10
Deaths	China	Female	Age-standardized	Tuberculosis	Smoking	Rate	2014	0,07	0,05	0,09
Deaths	China	Female	Age-standardized	Tuberculosis	Smoking	Rate	2015	0,06	0,04	0,09
Deaths	China	Female	Age-standardized	Tuberculosis	Smoking	Rate	2016	0,06	0,04	0,08
Deaths	China	Female	Age-standardized	Tuberculosis	Smoking	Rate	2017	0,06	0,04	0,08
Deaths	China	Female	Age-standardized	Tuberculosis	Smoking	Rate	2018	0,05	0,03	0,07
Deaths	China	Female	Age-standardized	Tuberculosis	Smoking	Rate	2019	0,05	0,03	0,07
Deaths	China	Female	Age-standardized	Tuberculosis	Smoking	Rate	2020	0,04	0,03	0,06
Deaths	China	Female	Age-standardized	Tuberculosis	Smoking	Rate	2021	0,04	0,03	0,06
DALYs	China	Male	All ages	Tuberculosis	Smoking	Number	1990	1411709	998625	1832715
DALYs	China	Male	All ages	Tuberculosis	Smoking	Number	1991	1388864	1029579	1785780

DALYs	China	Male	All ages	Tuberculosis	Smoking	Number	1992	1341195	1008215	1744031
DALYs	China	Male	All ages	Tuberculosis	Smoking	Number	1993	1280338	1000040	1623663
DALYs	China	Male	All ages	Tuberculosis	Smoking	Number	1994	1215835	957920	1522589
DALYs	China	Male	All ages	Tuberculosis	Smoking	Number	1995	1159412	926336	1448601
DALYs	China	Male	All ages	Tuberculosis	Smoking	Number	1996	1102759	875121	1353613
DALYs	China	Male	All ages	Tuberculosis	Smoking	Number	1997	1041265	825378	1296404
DALYs	China	Male	All ages	Tuberculosis	Smoking	Number	1998	993298	788027	1239026
DALYs	China	Male	All ages	Tuberculosis	Smoking	Number	1999	961276	767176	1176563
DALYs	China	Male	All ages	Tuberculosis	Smoking	Number	2000	946124	749282	1167594
DALYs	China	Male	All ages	Tuberculosis	Smoking	Number	2001	918052	733977	1149559
DALYs	China	Male	All ages	Tuberculosis	Smoking	Number	2002	904297	716590	1118074
DALYs	China	Male	All ages	Tuberculosis	Smoking	Number	2003	891637	692984	1103097
DALYs	China	Male	All ages	Tuberculosis	Smoking	Number	2004	870099	681595	1066826
DALYs	China	Male	All ages	Tuberculosis	Smoking	Number	2005	814084	650451	993679
DALYs	China	Male	All ages	Tuberculosis	Smoking	Number	2006	737569	588346	879025
DALYs	China	Male	All ages	Tuberculosis	Smoking	Number	2007	687233	542679	823603
DALYs	China	Male	All ages	Tuberculosis	Smoking	Number	2008	653893	520361	793849
DALYs	China	Male	All ages	Tuberculosis	Smoking	Number	2009	614318	497398	755630
DALYs	China	Male	All ages	Tuberculosis	Smoking	Number	2010	578833	454926	708809
DALYs	China	Male	All ages	Tuberculosis	Smoking	Number	2011	553683	439523	683978
DALYs	China	Male	All ages	Tuberculosis	Smoking	Number	2012	530277	409538	654184
DALYs	China	Male	All ages	Tuberculosis	Smoking	Number	2013	500844	393901	613154
DALYs	China	Male	All ages	Tuberculosis	Smoking	Number	2014	478684	369224	612074
DALYs	China	Male	All ages	Tuberculosis	Smoking	Number	2015	467339	369336	591234
DALYs	China	Male	All ages	Tuberculosis	Smoking	Number	2016	456665	349829	581425
DALYs	China	Male	All ages	Tuberculosis	Smoking	Number	2017	436330	327754	577777
DALYs	China	Male	All ages	Tuberculosis	Smoking	Number	2018	418586	311503	549749
DALYs	China	Male	All ages	Tuberculosis	Smoking	Number	2019	404318	288817	553098
DALYs	China	Male	All ages	Tuberculosis	Smoking	Number	2020	394280	283478	540510
DALYs	China	Male	All ages	Tuberculosis	Smoking	Number	2021	383792	276095	540736
DALYs	China	Male	Age-standardized	Tuberculosis	Smoking	Rate	1990	295,59	209,51	384,54
DALYs	China	Male	Age-standardized	Tuberculosis	Smoking	Rate	1991	283,16	210,06	363,03
DALYs	China	Male	Age-standardized	Tuberculosis	Smoking	Rate	1992	266,18	199,85	349,26

DALYs	China	Male	Age-standardized	Tuberculosis	Smoking	Rate	1993	247,34	192,15	314,35
DALYs	China	Male	Age-standardized	Tuberculosis	Smoking	Rate	1994	228,34	180,29	286,90
DALYs	China	Male	Age-standardized	Tuberculosis	Smoking	Rate	1995	211,96	168,18	265,50
DALYs	China	Male	Age-standardized	Tuberculosis	Smoking	Rate	1996	196,13	154,40	241,20
DALYs	China	Male	Age-standardized	Tuberculosis	Smoking	Rate	1997	180,35	142,31	223,79
DALYs	China	Male	Age-standardized	Tuberculosis	Smoking	Rate	1998	167,51	133,07	209,07
DALYs	China	Male	Age-standardized	Tuberculosis	Smoking	Rate	1999	157,83	125,68	193,95
DALYs	China	Male	Age-standardized	Tuberculosis	Smoking	Rate	2000	151,59	119,73	188,64
DALYs	China	Male	Age-standardized	Tuberculosis	Smoking	Rate	2001	143,66	114,45	179,87
DALYs	China	Male	Age-standardized	Tuberculosis	Smoking	Rate	2002	137,95	109,09	170,67
DALYs	China	Male	Age-standardized	Tuberculosis	Smoking	Rate	2003	132,82	102,98	164,96
DALYs	China	Male	Age-standardized	Tuberculosis	Smoking	Rate	2004	126,52	98,83	155,15
DALYs	China	Male	Age-standardized	Tuberculosis	Smoking	Rate	2005	115,94	92,34	141,26
DALYs	China	Male	Age-standardized	Tuberculosis	Smoking	Rate	2006	101,86	81,02	121,75
DALYs	China	Male	Age-standardized	Tuberculosis	Smoking	Rate	2007	92,44	72,71	111,46
DALYs	China	Male	Age-standardized	Tuberculosis	Smoking	Rate	2008	85,81	68,33	104,42
DALYs	China	Male	Age-standardized	Tuberculosis	Smoking	Rate	2009	78,79	63,89	97,12
DALYs	China	Male	Age-standardized	Tuberculosis	Smoking	Rate	2010	72,64	57,04	89,21
DALYs	China	Male	Age-standardized	Tuberculosis	Smoking	Rate	2011	67,92	53,98	83,92
DALYs	China	Male	Age-standardized	Tuberculosis	Smoking	Rate	2012	63,57	49,22	78,50
DALYs	China	Male	Age-standardized	Tuberculosis	Smoking	Rate	2013	58,77	46,25	72,00
DALYs	China	Male	Age-standardized	Tuberculosis	Smoking	Rate	2014	54,88	42,29	70,37
DALYs	China	Male	Age-standardized	Tuberculosis	Smoking	Rate	2015	52,38	41,48	66,08
DALYs	China	Male	Age-standardized	Tuberculosis	Smoking	Rate	2016	49,97	38,36	63,28
DALYs	China	Male	Age-standardized	Tuberculosis	Smoking	Rate	2017	46,64	35,20	61,40
DALYs	China	Male	Age-standardized	Tuberculosis	Smoking	Rate	2018	43,71	32,66	57,26
DALYs	China	Male	Age-standardized	Tuberculosis	Smoking	Rate	2019	41,18	29,79	56,17
DALYs	China	Male	Age-standardized	Tuberculosis	Smoking	Rate	2020	39,23	28,24	53,75
DALYs	China	Male	Age-standardized	Tuberculosis	Smoking	Rate	2021	37,39	26,91	52,70
DALYs	China	Female	All ages	Tuberculosis	Smoking	Number	1990	101107	71639	139432
DALYs	China	Female	All ages	Tuberculosis	Smoking	Number	1991	98555	70998	134942
DALYs	China	Female	All ages	Tuberculosis	Smoking	Number	1992	94710	68219	129579
DALYs	China	Female	All ages	Tuberculosis	Smoking	Number	1993	89666	64247	119623

DALYs	China	Female	All ages	Tuberculosis	Smoking	Number	1994	84206	59984	113352
DALYs	China	Female	All ages	Tuberculosis	Smoking	Number	1995	78323	56760	104918
DALYs	China	Female	All ages	Tuberculosis	Smoking	Number	1996	72624	53867	98388
DALYs	China	Female	All ages	Tuberculosis	Smoking	Number	1997	66814	48851	89082
DALYs	China	Female	All ages	Tuberculosis	Smoking	Number	1998	62370	45852	83137
DALYs	China	Female	All ages	Tuberculosis	Smoking	Number	1999	59380	43692	78791
DALYs	China	Female	All ages	Tuberculosis	Smoking	Number	2000	57435	42200	76078
DALYs	China	Female	All ages	Tuberculosis	Smoking	Number	2001	54833	40219	72121
DALYs	China	Female	All ages	Tuberculosis	Smoking	Number	2002	53228	39472	69082
DALYs	China	Female	All ages	Tuberculosis	Smoking	Number	2003	51389	37082	66711
DALYs	China	Female	All ages	Tuberculosis	Smoking	Number	2004	48459	36131	64554
DALYs	China	Female	All ages	Tuberculosis	Smoking	Number	2005	43434	31180	57200
DALYs	China	Female	All ages	Tuberculosis	Smoking	Number	2006	38073	28379	50679
DALYs	China	Female	All ages	Tuberculosis	Smoking	Number	2007	34155	24793	44300
DALYs	China	Female	All ages	Tuberculosis	Smoking	Number	2008	31208	22814	41032
DALYs	China	Female	All ages	Tuberculosis	Smoking	Number	2009	28620	20600	38464
DALYs	China	Female	All ages	Tuberculosis	Smoking	Number	2010	26404	19119	34580
DALYs	China	Female	All ages	Tuberculosis	Smoking	Number	2011	24701	18145	32538
DALYs	China	Female	All ages	Tuberculosis	Smoking	Number	2012	23097	16523	30068
DALYs	China	Female	All ages	Tuberculosis	Smoking	Number	2013	21301	15154	28672
DALYs	China	Female	All ages	Tuberculosis	Smoking	Number	2014	19935	14335	27234
DALYs	China	Female	All ages	Tuberculosis	Smoking	Number	2015	19357	13220	26624
DALYs	China	Female	All ages	Tuberculosis	Smoking	Number	2016	18955	12851	26280
DALYs	China	Female	All ages	Tuberculosis	Smoking	Number	2017	17895	12366	24295
DALYs	China	Female	All ages	Tuberculosis	Smoking	Number	2018	17008	11555	23384
DALYs	China	Female	All ages	Tuberculosis	Smoking	Number	2019	16325	11025	22766
DALYs	China	Female	All ages	Tuberculosis	Smoking	Number	2020	15811	10316	22208
DALYs	China	Female	All ages	Tuberculosis	Smoking	Number	2021	15326	10255	22686
DALYs	China	Female	Age-standardized	Tuberculosis	Smoking	Rate	1990	22,71	16,10	31,50
DALYs	China	Female	Age-standardized	Tuberculosis	Smoking	Rate	1991	21,60	15,52	29,51
DALYs	China	Female	Age-standardized	Tuberculosis	Smoking	Rate	1992	20,25	14,64	27,86
DALYs	China	Female	Age-standardized	Tuberculosis	Smoking	Rate	1993	18,69	13,54	24,96
DALYs	China	Female	Age-standardized	Tuberculosis	Smoking	Rate	1994	17,07	12,24	22,93

DALYs	China	Female	Age-standardized	Tuberculosis	Smoking	Rate	1995	15,43	11,18	20,39
DALYs	China	Female	Age-standardized	Tuberculosis	Smoking	Rate	1996	13,91	10,32	18,70
DALYs	China	Female	Age-standardized	Tuberculosis	Smoking	Rate	1997	12,44	9,13	16,57
DALYs	China	Female	Age-standardized	Tuberculosis	Smoking	Rate	1998	11,27	8,28	14,93
DALYs	China	Female	Age-standardized	Tuberculosis	Smoking	Rate	1999	10,43	7,72	13,81
DALYs	China	Female	Age-standardized	Tuberculosis	Smoking	Rate	2000	9,86	7,23	13,06
DALYs	China	Female	Age-standardized	Tuberculosis	Smoking	Rate	2001	9,16	6,73	12,07
DALYs	China	Female	Age-standardized	Tuberculosis	Smoking	Rate	2002	8,66	6,38	11,20
DALYs	China	Female	Age-standardized	Tuberculosis	Smoking	Rate	2003	8,13	5,91	10,49
DALYs	China	Female	Age-standardized	Tuberculosis	Smoking	Rate	2004	7,42	5,51	9,85
DALYs	China	Female	Age-standardized	Tuberculosis	Smoking	Rate	2005	6,46	4,67	8,52
DALYs	China	Female	Age-standardized	Tuberculosis	Smoking	Rate	2006	5,47	4,08	7,28
DALYs	China	Female	Age-standardized	Tuberculosis	Smoking	Rate	2007	4,74	3,47	6,16
DALYs	China	Female	Age-standardized	Tuberculosis	Smoking	Rate	2008	4,21	3,08	5,49
DALYs	China	Female	Age-standardized	Tuberculosis	Smoking	Rate	2009	3,75	2,71	5,05
DALYs	China	Female	Age-standardized	Tuberculosis	Smoking	Rate	2010	3,38	2,44	4,42
DALYs	China	Female	Age-standardized	Tuberculosis	Smoking	Rate	2011	3,07	2,26	4,01
DALYs	China	Female	Age-standardized	Tuberculosis	Smoking	Rate	2012	2,80	1,99	3,65
DALYs	China	Female	Age-standardized	Tuberculosis	Smoking	Rate	2013	2,51	1,78	3,38
DALYs	China	Female	Age-standardized	Tuberculosis	Smoking	Rate	2014	2,28	1,63	3,12
DALYs	China	Female	Age-standardized	Tuberculosis	Smoking	Rate	2015	2,15	1,47	2,93
DALYs	China	Female	Age-standardized	Tuberculosis	Smoking	Rate	2016	2,04	1,38	2,82
DALYs	China	Female	Age-standardized	Tuberculosis	Smoking	Rate	2017	1,87	1,30	2,54
DALYs	China	Female	Age-standardized	Tuberculosis	Smoking	Rate	2018	1,73	1,16	2,37
DALYs	China	Female	Age-standardized	Tuberculosis	Smoking	Rate	2019	1,61	1,08	2,23
DALYs	China	Female	Age-standardized	Tuberculosis	Smoking	Rate	2020	1,51	1,00	2,12
DALYs	China	Female	Age-standardized	Tuberculosis	Smoking	Rate	2021	1,43	0,96	2,10

TB, tuberculosis; DALYs, disability adjusted life years; ASRs, age-standardized rates; CI, confidence interval.

S8. Projected numbers of deaths and DALYs and their ASRs due to smoking-attributable TB in China, 2022-2040.

measure	location	sex	age	cause	rei	metric	year	pred_val	pred_low	pred_up
Deaths	China	Male	All ages	Tuberculosis	Smoking	Number	2022	10243	9259	11227
Deaths	China	Male	All ages	Tuberculosis	Smoking	Number	2023	9991	8708	11274
Deaths	China	Male	All ages	Tuberculosis	Smoking	Number	2024	9755	8039	11470
Deaths	China	Male	All ages	Tuberculosis	Smoking	Number	2025	9533	7294	11773
Deaths	China	Male	All ages	Tuberculosis	Smoking	Number	2026	9328	6498	12158
Deaths	China	Male	All ages	Tuberculosis	Smoking	Number	2027	9138	5661	12614
Deaths	China	Male	All ages	Tuberculosis	Smoking	Number	2028	8970	4793	13148
Deaths	China	Male	All ages	Tuberculosis	Smoking	Number	2029	8830	3893	13768
Deaths	China	Male	All ages	Tuberculosis	Smoking	Number	2030	8715	2952	14478
Deaths	China	Male	All ages	Tuberculosis	Smoking	Number	2031	8623	1958	15287
Deaths	China	Male	All ages	Tuberculosis	Smoking	Number	2032	8554	897	16210
Deaths	China	Male	All ages	Tuberculosis	Smoking	Number	2033	8514	0	17278
Deaths	China	Male	All ages	Tuberculosis	Smoking	Number	2034	8509	0	18532
Deaths	China	Male	All ages	Tuberculosis	Smoking	Number	2035	8539	0	20011
Deaths	China	Male	All ages	Tuberculosis	Smoking	Number	2036	8607	0	21769
Deaths	China	Male	All ages	Tuberculosis	Smoking	Number	2037	8714	0	23873
Deaths	China	Male	All ages	Tuberculosis	Smoking	Number	2038	8869	0	26434
Deaths	China	Male	All ages	Tuberculosis	Smoking	Number	2039	9081	0	29596
Deaths	China	Male	All ages	Tuberculosis	Smoking	Number	2040	9357	0	33535
Deaths	China	Male	Age-standardized	Tuberculosis	Smoking	Rate	2022	0,99	0,94	1,04
Deaths	China	Male	Age-standardized	Tuberculosis	Smoking	Rate	2023	0,94	0,85	1,04
Deaths	China	Male	Age-standardized	Tuberculosis	Smoking	Rate	2024	0,90	0,76	1,04
Deaths	China	Male	Age-standardized	Tuberculosis	Smoking	Rate	2025	0,85	0,67	1,04
Deaths	China	Male	Age-standardized	Tuberculosis	Smoking	Rate	2026	0,81	0,58	1,05
Deaths	China	Male	Age-standardized	Tuberculosis	Smoking	Rate	2027	0,78	0,49	1,06
Deaths	China	Male	Age-standardized	Tuberculosis	Smoking	Rate	2028	0,74	0,41	1,07
Deaths	China	Male	Age-standardized	Tuberculosis	Smoking	Rate	2029	0,71	0,33	1,08
Deaths	China	Male	Age-standardized	Tuberculosis	Smoking	Rate	2030	0,67	0,25	1,09
Deaths	China	Male	Age-standardized	Tuberculosis	Smoking	Rate	2031	0,64	0,18	1,11
Deaths	China	Male	Age-standardized	Tuberculosis	Smoking	Rate	2032	0,61	0,11	1,12
Deaths	China	Male	Age-standardized	Tuberculosis	Smoking	Rate	2033	0,58	0,04	1,13

Deaths	China	Male	Age-standardized	Tuberculosis	Smoking	Rate	2034	0,56	-0,03	1,14
Deaths	China	Male	Age-standardized	Tuberculosis	Smoking	Rate	2035	0,53	-0,09	1,15
Deaths	China	Male	Age-standardized	Tuberculosis	Smoking	Rate	2036	0,51	-0,14	1,16
Deaths	China	Male	Age-standardized	Tuberculosis	Smoking	Rate	2037	0,49	-0,20	1,17
Deaths	China	Male	Age-standardized	Tuberculosis	Smoking	Rate	2038	0,47	-0,25	1,18
Deaths	China	Male	Age-standardized	Tuberculosis	Smoking	Rate	2039	0,44	-0,29	1,18
Deaths	China	Male	Age-standardized	Tuberculosis	Smoking	Rate	2040	0,43	-0,34	1,19
Deaths	China	Female	All ages	Tuberculosis	Smoking	Number	2022	430	273	587
Deaths	China	Female	All ages	Tuberculosis	Smoking	Number	2023	415	250	580
Deaths	China	Female	All ages	Tuberculosis	Smoking	Number	2024	401	223	581
Deaths	China	Female	All ages	Tuberculosis	Smoking	Number	2025	389	191	587
Deaths	China	Female	All ages	Tuberculosis	Smoking	Number	2026	378	157	600
Deaths	China	Female	All ages	Tuberculosis	Smoking	Number	2027	368	120	619
Deaths	China	Female	All ages	Tuberculosis	Smoking	Number	2028	360	79	643
Deaths	China	Female	All ages	Tuberculosis	Smoking	Number	2029	353	37	675
Deaths	China	Female	All ages	Tuberculosis	Smoking	Number	2030	349	0	715
Deaths	China	Female	All ages	Tuberculosis	Smoking	Number	2031	346	0	765
Deaths	China	Female	All ages	Tuberculosis	Smoking	Number	2032	345	0	828
Deaths	China	Female	All ages	Tuberculosis	Smoking	Number	2033	347	0	907
Deaths	China	Female	All ages	Tuberculosis	Smoking	Number	2034	351	0	1009
Deaths	China	Female	All ages	Tuberculosis	Smoking	Number	2035	359	0	1141
Deaths	China	Female	All ages	Tuberculosis	Smoking	Number	2036	370	0	1313
Deaths	China	Female	All ages	Tuberculosis	Smoking	Number	2037	384	0	1538
Deaths	China	Female	All ages	Tuberculosis	Smoking	Number	2038	403	0	1840
Deaths	China	Female	All ages	Tuberculosis	Smoking	Number	2039	427	0	2251
Deaths	China	Female	All ages	Tuberculosis	Smoking	Number	2040	458	0	2819
Deaths	China	Female	Age-standardized	Tuberculosis	Smoking	Rate	2022	0,04	0,03	0,04
Deaths	China	Female	Age-standardized	Tuberculosis	Smoking	Rate	2023	0,04	0,03	0,04
Deaths	China	Female	Age-standardized	Tuberculosis	Smoking	Rate	2024	0,03	0,02	0,04
Deaths	China	Female	Age-standardized	Tuberculosis	Smoking	Rate	2025	0,03	0,02	0,04
Deaths	China	Female	Age-standardized	Tuberculosis	Smoking	Rate	2026	0,03	0,02	0,04
Deaths	China	Female	Age-standardized	Tuberculosis	Smoking	Rate	2027	0,03	0,01	0,04
Deaths	China	Female	Age-standardized	Tuberculosis	Smoking	Rate	2028	0,03	0,01	0,04

Deaths	China	Female	Age-standardized	Tuberculosis	Smoking	Rate	2029	0,02	0,01	0,04
Deaths	China	Female	Age-standardized	Tuberculosis	Smoking	Rate	2030	0,02	0,00	0,04
Deaths	China	Female	Age-standardized	Tuberculosis	Smoking	Rate	2031	0,02	0,00	0,04
Deaths	China	Female	Age-standardized	Tuberculosis	Smoking	Rate	2032	0,02	0,00	0,04
Deaths	China	Female	Age-standardized	Tuberculosis	Smoking	Rate	2033	0,02	-0,01	0,04
Deaths	China	Female	Age-standardized	Tuberculosis	Smoking	Rate	2034	0,02	-0,01	0,04
Deaths	China	Female	Age-standardized	Tuberculosis	Smoking	Rate	2035	0,02	-0,01	0,04
Deaths	China	Female	Age-standardized	Tuberculosis	Smoking	Rate	2036	0,02	-0,01	0,04
Deaths	China	Female	Age-standardized	Tuberculosis	Smoking	Rate	2037	0,01	-0,01	0,04
Deaths	China	Female	Age-standardized	Tuberculosis	Smoking	Rate	2038	0,01	-0,01	0,04
Deaths	China	Female	Age-standardized	Tuberculosis	Smoking	Rate	2039	0,01	-0,02	0,04
Deaths	China	Female	Age-standardized	Tuberculosis	Smoking	Rate	2040	0,01	-0,02	0,04
DALYs	China	Male	All ages	Tuberculosis	Smoking	Number	2022	368971	326117	411826
DALYs	China	Male	All ages	Tuberculosis	Smoking	Number	2023	356995	307896	406094
DALYs	China	Male	All ages	Tuberculosis	Smoking	Number	2024	345368	286285	404452
DALYs	China	Male	All ages	Tuberculosis	Smoking	Number	2025	334154	262322	405987
DALYs	China	Male	All ages	Tuberculosis	Smoking	Number	2026	323422	236924	409921
DALYs	China	Male	All ages	Tuberculosis	Smoking	Number	2027	313235	210695	415775
DALYs	China	Male	All ages	Tuberculosis	Smoking	Number	2028	303704	184044	423363
DALYs	China	Male	All ages	Tuberculosis	Smoking	Number	2029	294860	157135	432586
DALYs	China	Male	All ages	Tuberculosis	Smoking	Number	2030	286668	129960	443376
DALYs	China	Male	All ages	Tuberculosis	Smoking	Number	2031	279120	102436	455804
DALYs	China	Male	All ages	Tuberculosis	Smoking	Number	2032	272222	74405	470038
DALYs	China	Male	All ages	Tuberculosis	Smoking	Number	2033	266072	45664	486481
DALYs	China	Male	All ages	Tuberculosis	Smoking	Number	2034	260721	15989	505558
DALYs	China	Male	All ages	Tuberculosis	Smoking	Number	2035	256122	0	527624
DALYs	China	Male	All ages	Tuberculosis	Smoking	Number	2036	252251	0	553174
DALYs	China	Male	All ages	Tuberculosis	Smoking	Number	2037	249098	0	582848
DALYs	China	Male	All ages	Tuberculosis	Smoking	Number	2038	246724	0	617612
DALYs	China	Male	All ages	Tuberculosis	Smoking	Number	2039	245214	0	658738
DALYs	China	Male	All ages	Tuberculosis	Smoking	Number	2040	244618	0	707722
DALYs	China	Male	Age-standardized	Tuberculosis	Smoking	Rate	2022	35,01	32,95	37,07
DALYs	China	Male	Age-standardized	Tuberculosis	Smoking	Rate	2023	33,31	30,21	36,42

DALYs	China	Male	Age-standardized	Tuberculosis	Smoking	Rate	2024	31,70	27,36	36,04
DALYs	China	Male	Age-standardized	Tuberculosis	Smoking	Rate	2025	30,18	24,52	35,84
DALYs	China	Male	Age-standardized	Tuberculosis	Smoking	Rate	2026	28,74	21,73	35,75
DALYs	China	Male	Age-standardized	Tuberculosis	Smoking	Rate	2027	27,37	19,01	35,74
DALYs	China	Male	Age-standardized	Tuberculosis	Smoking	Rate	2028	26,08	16,38	35,78
DALYs	China	Male	Age-standardized	Tuberculosis	Smoking	Rate	2029	24,86	13,85	35,86
DALYs	China	Male	Age-standardized	Tuberculosis	Smoking	Rate	2030	23,69	11,43	35,96
DALYs	China	Male	Age-standardized	Tuberculosis	Smoking	Rate	2031	22,60	9,12	36,07
DALYs	China	Male	Age-standardized	Tuberculosis	Smoking	Rate	2032	21,55	6,93	36,18
DALYs	China	Male	Age-standardized	Tuberculosis	Smoking	Rate	2033	20,57	4,84	36,29
DALYs	China	Male	Age-standardized	Tuberculosis	Smoking	Rate	2034	19,63	2,87	36,39
DALYs	China	Male	Age-standardized	Tuberculosis	Smoking	Rate	2035	18,74	1,00	36,48
DALYs	China	Male	Age-standardized	Tuberculosis	Smoking	Rate	2036	17,90	-0,75	36,55
DALYs	China	Male	Age-standardized	Tuberculosis	Smoking	Rate	2037	17,10	-2,41	36,60
DALYs	China	Male	Age-standardized	Tuberculosis	Smoking	Rate	2038	16,34	-3,97	36,64
DALYs	China	Male	Age-standardized	Tuberculosis	Smoking	Rate	2039	15,61	-5,42	36,65
DALYs	China	Male	Age-standardized	Tuberculosis	Smoking	Rate	2040	14,92	-6,79	36,64
DALYs	China	Female	All ages	Tuberculosis	Smoking	Number	2022	14648	11923	17373
DALYs	China	Female	All ages	Tuberculosis	Smoking	Number	2023	14115	11162	17069
DALYs	China	Female	All ages	Tuberculosis	Smoking	Number	2024	13609	10265	16952
DALYs	China	Female	All ages	Tuberculosis	Smoking	Number	2025	13127	9261	16994
DALYs	China	Female	All ages	Tuberculosis	Smoking	Number	2026	12672	8180	17163
DALYs	China	Female	All ages	Tuberculosis	Smoking	Number	2027	12243	7049	17437
DALYs	China	Female	All ages	Tuberculosis	Smoking	Number	2028	11846	5885	17808
DALYs	China	Female	All ages	Tuberculosis	Smoking	Number	2029	11485	4694	18275
DALYs	China	Female	All ages	Tuberculosis	Smoking	Number	2030	11159	3475	18843
DALYs	China	Female	All ages	Tuberculosis	Smoking	Number	2031	10870	2218	19521
DALYs	China	Female	All ages	Tuberculosis	Smoking	Number	2032	10618	914	20328
DALYs	China	Female	All ages	Tuberculosis	Smoking	Number	2033	10409	0	21298
DALYs	China	Female	All ages	Tuberculosis	Smoking	Number	2034	10247	0	22467
DALYs	China	Female	All ages	Tuberculosis	Smoking	Number	2035	10127	0	23869
DALYs	China	Female	All ages	Tuberculosis	Smoking	Number	2036	10049	0	25553
DALYs	China	Female	All ages	Tuberculosis	Smoking	Number	2037	10012	0	27585

DALYs	China	Female	All ages	Tuberculosis	Smoking	Number	2038	10021	0	30062
DALYs	China	Female	All ages	Tuberculosis	Smoking	Number	2039	10083	0	33118
DALYs	China	Female	All ages	Tuberculosis	Smoking	Number	2040	10203	0	36923
DALYs	China	Female	Age-standardized	Tuberculosis	Smoking	Rate	2022	1,32	1,21	1,43
DALYs	China	Female	Age-standardized	Tuberculosis	Smoking	Rate	2023	1,24	1,09	1,40
DALYs	China	Female	Age-standardized	Tuberculosis	Smoking	Rate	2024	1,17	0,96	1,38
DALYs	China	Female	Age-standardized	Tuberculosis	Smoking	Rate	2025	1,11	0,84	1,37
DALYs	China	Female	Age-standardized	Tuberculosis	Smoking	Rate	2026	1,04	0,72	1,36
DALYs	China	Female	Age-standardized	Tuberculosis	Smoking	Rate	2027	0,98	0,61	1,36
DALYs	China	Female	Age-standardized	Tuberculosis	Smoking	Rate	2028	0,93	0,50	1,36
DALYs	China	Female	Age-standardized	Tuberculosis	Smoking	Rate	2029	0,88	0,40	1,36
DALYs	China	Female	Age-standardized	Tuberculosis	Smoking	Rate	2030	0,83	0,31	1,36
DALYs	China	Female	Age-standardized	Tuberculosis	Smoking	Rate	2031	0,79	0,22	1,36
DALYs	China	Female	Age-standardized	Tuberculosis	Smoking	Rate	2032	0,74	0,13	1,36
DALYs	China	Female	Age-standardized	Tuberculosis	Smoking	Rate	2033	0,71	0,05	1,36
DALYs	China	Female	Age-standardized	Tuberculosis	Smoking	Rate	2034	0,67	-0,02	1,36
DALYs	China	Female	Age-standardized	Tuberculosis	Smoking	Rate	2035	0,63	-0,09	1,35
DALYs	China	Female	Age-standardized	Tuberculosis	Smoking	Rate	2036	0,60	-0,15	1,35
DALYs	China	Female	Age-standardized	Tuberculosis	Smoking	Rate	2037	0,57	-0,21	1,35
DALYs	China	Female	Age-standardized	Tuberculosis	Smoking	Rate	2038	0,54	-0,26	1,35
DALYs	China	Female	Age-standardized	Tuberculosis	Smoking	Rate	2039	0,51	-0,32	1,34
DALYs	China	Female	Age-standardized	Tuberculosis	Smoking	Rate	2040	0,49	-0,36	1,34

TB, tuberculosis; DALYs, disability adjusted life years; ASRs, age-standardized rates; CI, confidence interval.

S9. Sensitivity analysis (Nordpred) of projected numbers of deaths and DALYs and their ASRs due to smoking-attributable TB in China, 2022-2040.

measure	location	sex	age	cause	rei	metric	year	case
Deaths	China	Male	All ages	Tuberculosis	Smoking	Number	2022	10013
Deaths	China	Male	All ages	Tuberculosis	Smoking	Number	2023	9552
Deaths	China	Male	All ages	Tuberculosis	Smoking	Number	2024	9059
Deaths	China	Male	All ages	Tuberculosis	Smoking	Number	2025	8918
Deaths	China	Male	All ages	Tuberculosis	Smoking	Number	2026	8762
Deaths	China	Male	All ages	Tuberculosis	Smoking	Number	2027	8588
Deaths	China	Male	All ages	Tuberculosis	Smoking	Number	2028	8398
Deaths	China	Male	All ages	Tuberculosis	Smoking	Number	2029	8193
Deaths	China	Male	All ages	Tuberculosis	Smoking	Number	2030	8234
Deaths	China	Male	All ages	Tuberculosis	Smoking	Number	2031	8266
Deaths	China	Male	All ages	Tuberculosis	Smoking	Number	2032	8285
Deaths	China	Male	All ages	Tuberculosis	Smoking	Number	2033	8293
Deaths	China	Male	All ages	Tuberculosis	Smoking	Number	2034	8292
Deaths	China	Male	All ages	Tuberculosis	Smoking	Number	2035	8501
Deaths	China	Male	All ages	Tuberculosis	Smoking	Number	2036	8707
Deaths	China	Male	All ages	Tuberculosis	Smoking	Number	2037	8906
Deaths	China	Male	All ages	Tuberculosis	Smoking	Number	2038	9100
Deaths	China	Male	All ages	Tuberculosis	Smoking	Number	2039	9290
Deaths	China	Male	All ages	Tuberculosis	Smoking	Number	2040	9472
Deaths	China	Male	Age-standardized	Tuberculosis	Smoking	Rate	2022	0,96
Deaths	China	Male	Age-standardized	Tuberculosis	Smoking	Rate	2023	0,89
Deaths	China	Male	Age-standardized	Tuberculosis	Smoking	Rate	2024	0,83
Deaths	China	Male	Age-standardized	Tuberculosis	Smoking	Rate	2025	0,79
Deaths	China	Male	Age-standardized	Tuberculosis	Smoking	Rate	2026	0,76
Deaths	China	Male	Age-standardized	Tuberculosis	Smoking	Rate	2027	0,73
Deaths	China	Male	Age-standardized	Tuberculosis	Smoking	Rate	2028	0,70
Deaths	China	Male	Age-standardized	Tuberculosis	Smoking	Rate	2029	0,67
Deaths	China	Male	Age-standardized	Tuberculosis	Smoking	Rate	2030	0,66
Deaths	China	Male	Age-standardized	Tuberculosis	Smoking	Rate	2031	0,64
Deaths	China	Male	Age-standardized	Tuberculosis	Smoking	Rate	2032	0,63
Deaths	China	Male	Age-standardized	Tuberculosis	Smoking	Rate	2033	0,62
Deaths	China	Male	Age-standardized	Tuberculosis	Smoking	Rate	2034	0,61
Deaths	China	Male	Age-standardized	Tuberculosis	Smoking	Rate	2035	0,62
Deaths	China	Male	Age-standardized	Tuberculosis	Smoking	Rate	2036	0,62
Deaths	China	Male	Age-standardized	Tuberculosis	Smoking	Rate	2037	0,63
Deaths	China	Male	Age-standardized	Tuberculosis	Smoking	Rate	2038	0,63
Deaths	China	Male	Age-standardized	Tuberculosis	Smoking	Rate	2039	0,64
Deaths	China	Male	Age-standardized	Tuberculosis	Smoking	Rate	2040	0,64
Deaths	China	Female	All ages	Tuberculosis	Smoking	Number	2022	425
Deaths	China	Female	All ages	Tuberculosis	Smoking	Number	2023	402
Deaths	China	Female	All ages	Tuberculosis	Smoking	Number	2024	376
Deaths	China	Female	All ages	Tuberculosis	Smoking	Number	2025	372
Deaths	China	Female	All ages	Tuberculosis	Smoking	Number	2026	367
Deaths	China	Female	All ages	Tuberculosis	Smoking	Number	2027	361
Deaths	China	Female	All ages	Tuberculosis	Smoking	Number	2028	354
Deaths	China	Female	All ages	Tuberculosis	Smoking	Number	2029	346
Deaths	China	Female	All ages	Tuberculosis	Smoking	Number	2030	353

Deaths	China	Female	All ages	Tuberculosis	Smoking	Number	2031	359
Deaths	China	Female	All ages	Tuberculosis	Smoking	Number	2032	365
Deaths	China	Female	All ages	Tuberculosis	Smoking	Number	2033	371
Deaths	China	Female	All ages	Tuberculosis	Smoking	Number	2034	376
Deaths	China	Female	All ages	Tuberculosis	Smoking	Number	2035	395
Deaths	China	Female	All ages	Tuberculosis	Smoking	Number	2036	415
Deaths	China	Female	All ages	Tuberculosis	Smoking	Number	2037	435
Deaths	China	Female	All ages	Tuberculosis	Smoking	Number	2038	454
Deaths	China	Female	All ages	Tuberculosis	Smoking	Number	2039	474
Deaths	China	Female	All ages	Tuberculosis	Smoking	Number	2040	493
Deaths	China	Female	Age-standardized	Tuberculosis	Smoking	Rate	2022	0,04
Deaths	China	Female	Age-standardized	Tuberculosis	Smoking	Rate	2023	0,03
Deaths	China	Female	Age-standardized	Tuberculosis	Smoking	Rate	2024	0,03
Deaths	China	Female	Age-standardized	Tuberculosis	Smoking	Rate	2025	0,03
Deaths	China	Female	Age-standardized	Tuberculosis	Smoking	Rate	2026	0,03
Deaths	China	Female	Age-standardized	Tuberculosis	Smoking	Rate	2027	0,03
Deaths	China	Female	Age-standardized	Tuberculosis	Smoking	Rate	2028	0,03
Deaths	China	Female	Age-standardized	Tuberculosis	Smoking	Rate	2029	0,03
Deaths	China	Female	Age-standardized	Tuberculosis	Smoking	Rate	2030	0,03
Deaths	China	Female	Age-standardized	Tuberculosis	Smoking	Rate	2031	0,02
Deaths	China	Female	Age-standardized	Tuberculosis	Smoking	Rate	2032	0,02
Deaths	China	Female	Age-standardized	Tuberculosis	Smoking	Rate	2033	0,02
Deaths	China	Female	Age-standardized	Tuberculosis	Smoking	Rate	2034	0,02
Deaths	China	Female	Age-standardized	Tuberculosis	Smoking	Rate	2035	0,03
Deaths	China	Female	Age-standardized	Tuberculosis	Smoking	Rate	2036	0,03
Deaths	China	Female	Age-standardized	Tuberculosis	Smoking	Rate	2037	0,03
Deaths	China	Female	Age-standardized	Tuberculosis	Smoking	Rate	2038	0,03
Deaths	China	Female	Age-standardized	Tuberculosis	Smoking	Rate	2039	0,03
Deaths	China	Female	Age-standardized	Tuberculosis	Smoking	Rate	2040	0,03
DALYs	China	Male	All ages	Tuberculosis	Smoking	Number	2022	368019
DALYs	China	Male	All ages	Tuberculosis	Smoking	Number	2023	350502
DALYs	China	Male	All ages	Tuberculosis	Smoking	Number	2024	331895
DALYs	China	Male	All ages	Tuberculosis	Smoking	Number	2025	323395
DALYs	China	Male	All ages	Tuberculosis	Smoking	Number	2026	314396
DALYs	China	Male	All ages	Tuberculosis	Smoking	Number	2027	304892
DALYs	China	Male	All ages	Tuberculosis	Smoking	Number	2028	294915
DALYs	China	Male	All ages	Tuberculosis	Smoking	Number	2029	284444
DALYs	China	Male	All ages	Tuberculosis	Smoking	Number	2030	281007
DALYs	China	Male	All ages	Tuberculosis	Smoking	Number	2031	277299
DALYs	China	Male	All ages	Tuberculosis	Smoking	Number	2032	273252
DALYs	China	Male	All ages	Tuberculosis	Smoking	Number	2033	268914
DALYs	China	Male	All ages	Tuberculosis	Smoking	Number	2034	264308
DALYs	China	Male	All ages	Tuberculosis	Smoking	Number	2035	265213
DALYs	China	Male	All ages	Tuberculosis	Smoking	Number	2036	265896
DALYs	China	Male	All ages	Tuberculosis	Smoking	Number	2037	266249
DALYs	China	Male	All ages	Tuberculosis	Smoking	Number	2038	266258
DALYs	China	Male	All ages	Tuberculosis	Smoking	Number	2039	265968
DALYs	China	Male	All ages	Tuberculosis	Smoking	Number	2040	265348
DALYs	China	Male	Age-standardized	Tuberculosis	Smoking	Rate	2022	34,82
DALYs	China	Male	Age-standardized	Tuberculosis	Smoking	Rate	2023	32,60

DALYs	China	Male	Age-standardized	Tuberculosis	Smoking	Rate	2024	30,37
DALYs	China	Male	Age-standardized	Tuberculosis	Smoking	Rate	2025	29,13
DALYs	China	Male	Age-standardized	Tuberculosis	Smoking	Rate	2026	27,88
DALYs	China	Male	Age-standardized	Tuberculosis	Smoking	Rate	2027	26,64
DALYs	China	Male	Age-standardized	Tuberculosis	Smoking	Rate	2028	25,40
DALYs	China	Male	Age-standardized	Tuberculosis	Smoking	Rate	2029	24,15
DALYs	China	Male	Age-standardized	Tuberculosis	Smoking	Rate	2030	23,54
DALYs	China	Male	Age-standardized	Tuberculosis	Smoking	Rate	2031	22,93
DALYs	China	Male	Age-standardized	Tuberculosis	Smoking	Rate	2032	22,32
DALYs	China	Male	Age-standardized	Tuberculosis	Smoking	Rate	2033	21,71
DALYs	China	Male	Age-standardized	Tuberculosis	Smoking	Rate	2034	21,10
DALYs	China	Male	Age-standardized	Tuberculosis	Smoking	Rate	2035	20,96
DALYs	China	Male	Age-standardized	Tuberculosis	Smoking	Rate	2036	20,81
DALYs	China	Male	Age-standardized	Tuberculosis	Smoking	Rate	2037	20,66
DALYs	China	Male	Age-standardized	Tuberculosis	Smoking	Rate	2038	20,52
DALYs	China	Male	Age-standardized	Tuberculosis	Smoking	Rate	2039	20,37
DALYs	China	Male	Age-standardized	Tuberculosis	Smoking	Rate	2040	20,22
DALYs	China	Female	All ages	Tuberculosis	Smoking	Number	2022	14599
DALYs	China	Female	All ages	Tuberculosis	Smoking	Number	2023	13769
DALYs	China	Female	All ages	Tuberculosis	Smoking	Number	2024	12871
DALYs	China	Female	All ages	Tuberculosis	Smoking	Number	2025	12583
DALYs	China	Female	All ages	Tuberculosis	Smoking	Number	2026	12268
DALYs	China	Female	All ages	Tuberculosis	Smoking	Number	2027	11923
DALYs	China	Female	All ages	Tuberculosis	Smoking	Number	2028	11549
DALYs	China	Female	All ages	Tuberculosis	Smoking	Number	2029	11146
DALYs	China	Female	All ages	Tuberculosis	Smoking	Number	2030	11131
DALYs	China	Female	All ages	Tuberculosis	Smoking	Number	2031	11103
DALYs	China	Female	All ages	Tuberculosis	Smoking	Number	2032	11060
DALYs	China	Female	All ages	Tuberculosis	Smoking	Number	2033	11002
DALYs	China	Female	All ages	Tuberculosis	Smoking	Number	2034	10930
DALYs	China	Female	All ages	Tuberculosis	Smoking	Number	2035	11204
DALYs	China	Female	All ages	Tuberculosis	Smoking	Number	2036	11476
DALYs	China	Female	All ages	Tuberculosis	Smoking	Number	2037	11741
DALYs	China	Female	All ages	Tuberculosis	Smoking	Number	2038	11998
DALYs	China	Female	All ages	Tuberculosis	Smoking	Number	2039	12246
DALYs	China	Female	All ages	Tuberculosis	Smoking	Number	2040	12480
DALYs	China	Female	Age-standardized	Tuberculosis	Smoking	Rate	2022	1,31
DALYs	China	Female	Age-standardized	Tuberculosis	Smoking	Rate	2023	1,21
DALYs	China	Female	Age-standardized	Tuberculosis	Smoking	Rate	2024	1,11
DALYs	China	Female	Age-standardized	Tuberculosis	Smoking	Rate	2025	1,06
DALYs	China	Female	Age-standardized	Tuberculosis	Smoking	Rate	2026	1,01
DALYs	China	Female	Age-standardized	Tuberculosis	Smoking	Rate	2027	0,97
DALYs	China	Female	Age-standardized	Tuberculosis	Smoking	Rate	2028	0,92
DALYs	China	Female	Age-standardized	Tuberculosis	Smoking	Rate	2029	0,87
DALYs	China	Female	Age-standardized	Tuberculosis	Smoking	Rate	2030	0,85
DALYs	China	Female	Age-standardized	Tuberculosis	Smoking	Rate	2031	0,84
DALYs	China	Female	Age-standardized	Tuberculosis	Smoking	Rate	2032	0,82
DALYs	China	Female	Age-standardized	Tuberculosis	Smoking	Rate	2033	0,81
DALYs	China	Female	Age-standardized	Tuberculosis	Smoking	Rate	2034	0,79
DALYs	China	Female	Age-standardized	Tuberculosis	Smoking	Rate	2035	0,80

DALYs	China	Female	Age-standardized	Tuberculosis	Smoking	Rate	2036	0,81
DALYs	China	Female	Age-standardized	Tuberculosis	Smoking	Rate	2037	0,82
DALYs	China	Female	Age-standardized	Tuberculosis	Smoking	Rate	2038	0,83
DALYs	China	Female	Age-standardized	Tuberculosis	Smoking	Rate	2039	0,84
DALYs	China	Female	Age-standardized	Tuberculosis	Smoking	Rate	2040	0,85

TB, tuberculosis; DALYs, disability adjusted life years; ASRs, age-standardized rates.

S10. Decomposition analysis of the contribution of population aging, population growth and epidemiological changes to smoking-attributable TB deaths and DALYs in China, 1990-2021.

measure	location	sex	cause	rei	Overll difference	Aging	Population	Epidemiological change
Deaths	China	Both	Tuberculosis	Smoking	-33311.20 (-75.44%)	9446.43 (21.39%)	24118.95 (54.62%)	-66876.58 (-151.45%)
Deaths	China	Male	Tuberculosis	Smoking	-30294.29 (-74.45%)	9551.73 (23.48%)	21313.84 (52.38%)	-61159.86 (-150.31%)
Deaths	China	Female	Tuberculosis	Smoking	-3016.90 (-87.00%)	986.14 (28.44%)	1970.48 (56.83%)	-5973.52 (-172.27%)
DALYs	China	Both	Tuberculosis	Smoking	-1113698.64 (-73.62%)	184364.63 (12.19%)	806010.68 (53.28%)	-2104073.95 (-139.08%)
DALYs	China	Male	Tuberculosis	Smoking	-1027917.39 (-72.81%)	185527.58 (13.14%)	717982.76 (50.86%)	-1931427.73 (-136.81%)
DALYs	China	Female	Tuberculosis	Smoking	-85781.25 (-84.84%)	17955.55 (17.76%)	55563.38 (54.95%)	-159300.18 (-157.56%)

TB, tuberculosis; DALYs, disability adjusted life years.
